# Assessment of candidate high-grade serous ovarian carcinoma predisposition genes through integrated germline and tumour sequencing

**DOI:** 10.1038/s41525-024-00447-3

**Published:** 2025-01-10

**Authors:** Deepak N. Subramanian, Maia Zethoven, Kathleen I. Pishas, Evanny R. Marinović, Simone McInerny, Simone M. Rowley, Prue E. Allan, Lisa Devereux, Dane Cheasley, Paul A. James, Ian G. Campbell

**Affiliations:** 1 Cancer Genetics Laboratory, Peter MacCallum Cancer Centre, Melbourne, VIC Australia; 2 Sir Peter MacCallum Department of Oncology, The University of Melbourne, Melbourne, VIC Australia; 3 Bioinformatics Core Facility, Peter MacCallum Cancer Centre, Melbourne, VIC Australia; 4 Parkville Familial Cancer Centre, Peter MacCallum Cancer Centre and The Royal Melbourne Hospital, Melbourne, VIC Australia; 5 Department of Pathology, Peter MacCallum Cancer Centre, Melbourne, VIC Australia; 6 Lifepool Cohort, Peter MacCallum Cancer Centre, Melbourne, VIC Australia

**Keywords:** Ovarian cancer, Genetics research, Cancer genetics, Cancer genomics

## Abstract

High-grade serous ovarian carcinoma (HGSOC) has a significant hereditary component, only half of which is explained. Previously, we performed germline exome sequencing on *BRCA1* and *BRCA2*-negative HGSOC patients, revealing three proposed and 43 novel candidate genes enriched with rare loss-of-function variants. For validation, we undertook case-control analyses using genomic data from disease-free controls. This confirms enrichment for nearly all previously identified genes. Additionally, one-hundred-and-eleven HGSOC tumours from variant carriers were sequenced alongside other complementary studies, seeking evidence of biallelic inactivation as supportive evidence. *PALB2* and *ATM* validate as HGSOC predisposition genes, with 6/8 germline carrier tumours exhibiting biallelic inactivation accompanied by characteristic mutational signatures. Among candidate genes, only *LLGL2* consistently shows biallelic inactivation and protein expression loss, supporting it as a novel HGSOC susceptibility gene. The remaining candidate genes fail to validate. Integrating case-control analyses with tumour sequencing is thus crucial for accurate gene discovery in familial cancer studies.

## Introduction

High-grade serous ovarian carcinoma (HGSOC) is the most prevalent epithelial ovarian tumour type, often diagnosed at an advanced stage with associated high morbidity and mortality^1^. Approximately 40% has a significant hereditary component^2,3^, of which only ~50% can be explained by germline pathogenic variants in the known hereditary breast and ovarian cancer (HBOC) genes *BRCA1, BRCA2, RAD51C, RAD51D* and *BRIP1*^3^. Although recent data supports a modest increased risk for loss-of-function (LoF) variants in *PALB2*^4,5^ and *ATM*^6^, efforts to identify other high-risk ovarian cancer predisposition genes have been largely unsuccessful.

Previously, germline whole exome sequencing (WES) was performed on 516 women from the Variants in Practice (ViP) cohort with HGSOC of suspected familial origin where clinical genetic testing did not identify any pathogenic variants in *BRCA1* or *BRCA2*^7^. This analysis identified 1307 genes enriched for rare, protein-coding germline LoF variants compared to the gnomAD cancer-free control population^8^, but a high degree of genetic heterogeneity was observed with no individual gene found to harbour LoF variants in more than 2.4% of cases. In addition to potentially pathogenic variants in known and proposed HGSOC predisposition genes, LoF variants were identified in 43 novel and functionally diverse candidate genes, few of which are involved in DNA repair as is the case with the established HBOC genes. However, as the number of cases for each candidate gene was small, it was not possible to confidently promote any of these as HGSOC predisposition genes. Hence, orthogonal experimental approaches are required to corroborate these findings.

One approach previously used to validate candidate predisposition genes (e.g. *PALB2*^9^) is to sequence tumours from germline variant carriers to look for evidence of biallelic inactivation. This is consistently observed for the HBOC genes identified to date^10–12^, although not all novel cancer predisposition genes may act via this ‘two-hit’ mechanism^13^. Tumour sequencing data can additionally be interrogated for somatic genomic features that are consistent with loss of activity of the candidate gene, including homologous recombination deficiency (HRD)^14^ and mutational signatures^15^. Integration of somatic genetic data with germline data can impart powerful insights into candidate genes and variants, providing either supportive or invalidating evidence for their role in tumourigenesis, as shown previously by us and others for breast tumours^16–24^.

Here, we employ this approach using HGSOC tumours from 111 carriers of germline LoF variants in 43 candidate genes as well as six proposed predisposition genes. Furthermore, case-control analyses are extended using local disease-free controls from the Medical Genomics Reference Bank (MGRB)^25^ and Lifepool^26^ to corroborate our earlier findings. These analyses provide additional support for the roles of *PALB2* and *ATM* as HGSOC predisposition genes, alongside evidence for *LLGL2* as a potential novel susceptibility gene.

## Results

### LoF variant enrichment in HGSOC cases versus MGRB and Lifepool controls

Table 1 summarises the frequency in the discovery case cohort of rare LoF variants found in 43 candidate genes and six accepted or proposed HGSOC predisposition genes compared to the frequency among MGRB and Lifepool local control cohorts (separate and combined), alongside gnomAD^8^. The candidate genes were originally identified through comparison with the frequency in gnomAD, and for most genes (24/43 candidate genes, plus *PALB2* and *ATM*), the odds ratios for LoF variant enrichment in cases versus the combined local control cohort were lower than the equivalent figures observed versus gnomAD. Conversely, five genes (*WRAP53*, *LLGL2*, *CCDC14*, *TTC24*, *ZNF418*) showed increases in odds ratio for enrichment large enough to elevate their ranking into the top ten. Regardless, all genes except *SORD* and *FANCM* showed a minimum twofold higher excess in the cases versus the local controls, closely approximating the minimum near threefold excess observed versus gnomAD.

**Table 1:** Frequency of loss-of-function (LoF) variant-containing alleles in candidate and proposed genes in cases (*n* = 496^a^) versus controls, ordered by ascending *p*-value against gnomAD controls (’gnomAD rank’) for each category, alongside the number of available tumours for sequencing per gene

Gene	No. of LoF alleles in cases (AF%)	GnomAD controls^b^						No. of LoF alleles in MGRB controls^d^ (AF%)	No. of LoF alleles in Lifepool controls^f^ (AF%)	Combined MGRB and Lifepool controls^g^					No. of sequenced tumours
		No. of LoF alleles (AF%)	OR^c^		95% CI^c^	*P*-value^c^	Rank			No. of LoF alleles (AF%)	OR^c^	95% CI^c^	*P*-value^c^	Rank	
**Proposed genes**															
* PALB2*	3 (0.30)	86 (0.073)	4.2	0.8 to 12.6		0.039	41	4 (0.078)	3 (0.088)	7 (0.082)	3.7	0.6 to 16.3	0.077	38	3^h^
* ATM*	4 (0.40)^a^	195 (0.16)	2.4	0.7 to 6.4		0.086	43	13 (0.25)	3 (0.088)	16 (0.19)	2.1	0.5 to 6.7	0.15	44	5^i^
* MRE11A*	2 (0.20)^a^	57 (0.048)	4.2	0.5 to 15.9		0.087	44	1 (0.019)	2 (0.059)	3 (0.035)	5.6	0.5 to 48.8	0.091	40	2
* ERCC3*	3 (0.30)	125 (0.11)	2.9	0.6 to 8.6		0.092	45	2 (0.039)	2 (0.059)	4 (0.047)	6.5	1.0 to 38.3	0.028	29	3
* BLM*	3 (0.30)	131 (0.11)	2.7	0.6 to 8.2		0.10	46	5 (0.097)	3 (0.088)	8 (0.094)	3.2	0.6 to 13.5	0.098	41	3
* FANCM*	2 (0.20)^a^	344 (0.29)	0.7	0.1 to 2.5		1.0	49	11 (0.21)	7 (0.21)	18 (0.21)	0.94	0.1 to 3.9	1.0	49	2
**Candidate genes**															
* MAP6D1*	3 (0.30)	9 (0.0076)	39.8	6.9 to 159.2		0.00012	1	3 (0.058)	1 (0.029)	4 (0.047)	6.5	0.9 to 38.2	0.029	32	3
* SLC12A4*	5 (0.50)^a^	54 (0.046)	11.1	3.5 to 27.6		0.00013	2	6 (0.12)	0 (0)	6 (0.070)	7.2	1.7 to 28.4	0.0032	6	5
* SORD*	6 (0.60)	89 (0.075)	8.1	2.9 to 18.3		0.00015	3	26 (0.51)	0 (0)	26 (0.30)	2.0	0.7 to 4.9	0.14	43	4
* CPT1B*	4 (0.40)	36 (0.030)	13.3	3.4 to 37.1		0.00034	4	2 (0.039)	0 (0)	2 (0.023)	17.3	2.5 to 191.1	0.0015	2	2
* ZBTB45*	4 (0.40)	37 (0.031)	12.9	3.3 to 36.1		0.00038	5	3 (0.058)	0 (0)	3 (0.035)	11.5	2.0 to 78.8	0.0031	5	4
* LOXL2*	4 (0.40)	39 (0.033)	12.3	3.2 to 34.1		0.00045	6	3 (0.058)	2 (0.059)	5 (0.058)	6.9	1.34 to 32.2	0.0095	17	3
* SSX3*	3 (0.30)	11 (0.013)	23.2	4.2 to 88.2		0.00052	7	1 (0.019)^e^	0 (0)	1 (0.012)	25.9	2.1 to 1349.4	0.0041	9	2
* ZCCHC4*	12 (1.2)	444 (0.38)	3.2	1.7 to 5.7		0.00055	8	20 (0.39)	8 (0.23)	28 (0.33)	3.7	1.7 to 7.6	0.00053	1	10
* RPA3*	3 (0.30)	18 (0.015)	19.9	3.8 to 68.3		0.00068	9	2 (0.039)	1 (0.029)	3 (0.035)	8.6	1.2 to 64.7	0.018	23	2
* IMPDH2*	3 (0.30)	19 (0.016)	18.9	3.6 to 64.2		0.00079	10	1 (0.019)	0 (0)	1 (0.012)	25.9	2.1 to 1348.8	0.0041	11	3
* GPALPP1*	3 (0.30)	19 (0.016)	18.8	3.6 to 64.1		0.00079	11	2 (0.039)	0 (0)	2 (0.023)	13.0	1.5 to 155.2	0.0095	15	3
* WRAP53*	4 (0.40)	49 (0.041)	9.8	2.6 to 26.7		0.0010	12	2 (0.039)	1 (0.029)	3 (0.035)	11.5	2.0 to 78.8	0.0031	3	4
* STARD6*	3 (0.30)	22 (0.019)	16.3	3.1 to 54.4		0.0012	13	0 (0)	2 (0.059)	2 (0.023)	12.8	1.5 to 153.8	0.0098	20	2
* LLGL2*	4 (0.40)	55 (0.047)	8.7	2.3 to 23.6		0.0015	14	0 (0)	3 (0.088)	3 (0.035)	11.5	2.0 to 78.8	0.0031	4	3
* CCDC88B*	4 (0.40)^a^	57 (0.048)	8.4	2.2 to 22.7		0.0017	15	2 (0.039)	4 (0.12)	6 (0.070)	5.8	1.2 to 24.3	0.015	22	3
* FBLIM1*	3 (0.30)	26 (0.022)	13.8	2.7 to 45.0		0.0018	16	2 (0.039)	0 (0)	2 (0.023)	12.9	1.5 to 155.1	0.0096	19	2
* IFIT2*	4 (0.40)	58 (0.049)	8.2	2.2 to 22.2		0.0018	17	3 (0.058)	1 (0.029)	4 (0.047)	8.6	1.6 to 46.5	0.0058	12	3
* MIPOL1*	4 (0.40)	60 (0.051)	8.0	2.1 to 21.5		0.0020	18	6 (0.12)	3 (0.088)	9 (0.11)	3.3	0.7 to 11.9	0.059	36	3
* CCDC14*	6 (0.60)	150 (0.13)	4.8	1.7 to 10.7		0.0021	19	9 (0.17)	1 (0.029)	10 (0.12)	5.2	1.6 to 15.8	0.0040	8	6
* TTC24*	3 (0.30)	32 (0.027)	11.1	2.2 to 35.7		0.0031	20	0 (0)	1 (0.029)	1 (0.012)	25.9	2.1 to 1349.4	0.0041	10	2
* SLC38A8*	3 (0.30)	34 (0.029)	10.5	2.1 to 33.6		0.0036	21	2 (0.039)	2 (0.059)	4 (0.047)	6.5	1.0 to 38.3	0.028	27	2
* ANKAR*	6 (0.60)	174 (0.15)	4.1	1.5 to 9.2		0.0042	22	8 (0.16)	11 (0.32)	19 (0.22)	2.7	0.9 to 7.1	0.041	33	5
* CARMIL2*	3 (0.30)	36 (0.030)	9.9	2.0 to 31.4		0.0043	23	3 (0.058)	1 (0.029)	4 (0.047)	6.5	1.0 to 38.3	0.028	31	2
* SCYL3*	3 (0.30)	37 (0.031)	9.7	1.9 to 30.7		0.0045	24	6 (0.12)	1 (0.029)	7 (0.082)	3.7	0.6 to 16.3	0.077	37	3
* MMAA*	4 (0.40)^a^	78 (0.066)	6.1	1.6 to 16.4		0.0050	25	4 (0.078)	3 (0.088)	7 (0.082)	4.9	1.1 to 19.3	0.022	26	2
* ZNF418*	5 (0.50)	127 (0.11)	4.7	1.5 to 11.3		0.0051	26	3 (0.058)	3 (0.088)	6 (0.070)	7.2	1.7 to 28.4	0.0033	7	2
* RAD1*	5 (0.50)	129 (0.11)	4.6	1.5 to 11.1		0.0055	27	6 (0.12)	2 (0.059)	8 (0.094)	5.4	1.4 to 18.8	0.0076	13	3
* USP50*	4 (0.40)	80 (0.068)	5.9	1.6 to 15.9		0.0055	28	2 (0.039)	3 (0.088)	5 (0.058)	6.9	1.4 to 32.2	0.0095	14	4
* RASSF7*	3 (0.30)	41 (0.035)	8.7	1.7 to 27.4		0.0059	29	3 (0.058)	1 (0.029)	4 (0.047)	6.5	1.0 to 38.3	0.028	30	3
* LRRC56*	3 (0.30)	44 (0.037)	8.1	1.6 to 25.5		0.0071	30	1 (0.019)	7 (0.21)	8 (0.094)	3.2	0.6 to 13.5	0.098	42	1
* HARS2*	3 (0.30)	45 (0.038)	8.0	1.6 to 24.9		0.0075	31	2 (0.039)	0 (0)	2 (0.023)	13.0	1.5 to 155.2	0.0095	16	2
* PRKACG*	3 (0.30)	46 (0.039)	7.8	1.6 to 24.3		0.0080	32	0 (0)	6 (0.18)	6 (0.070)	4.3	0.7 to 20.3	0.058	35	2
* CDKL3*	4 (0.40)	90 (0.076)	5.3	1.4 to 14.0		0.0082	33	7 (0.14)	0 (0)	7 (0.082)	4.9	1.1 to 19.5	0.021	25	2
* ZNF616*	4 (0.40)	91 (0.077)	5.3	1.4 to 14.0		0.0084	34	4 (0.078)	2 (0.059)	6 (0.070)	5.8	1.2 to 24.3	0.015	21	3
* VSIG1*	3 (0.30)	33 (0.039)	7.7	1.5 to 24.8		0.0084	35	2 (0.039)^e^	0 (0)	2 (0.023)	13.0	1.5 to 155.2	0.0095	18	1
* ANKRD18A*	3 (0.30)	47 (0.040)	7.6	1.5 to 23.6		0.0085	36	0 (0)	3 (0.088)	3 (0.035)	8.5	1.1 to 63.3	0.019	24	3
* FAM216A*	3 (0.30)^a^	60 (0.051)	6.0	1.2 to 18.4		0.016	37	7 (0.14)	3 (0.088)	10 (0.12)	2.6	0.4 to 9.8	0.15	45	2
* TBXAS1*	3 (0.30)^a^	61 (0.052)	5.9	1.2 to 18.0		0.016	38	3 (0.058)	1 (0.029)	4 (0.047)	6.5	1.0 to 38.3	0.028	28	1
* CDH23*	2 (0.20)^a^	26 (0.022)	9.1	1.1 to 36.6		0.023	39	4 (0.078)	1 (0.029)	5 (0.058)	3.5	0.3 to 21.1	0.16	46	2
* LTBP1*	2 (0.20)^a^	28 (0.024)	8.5	1.0 to 33.9		0.026	40	3 (0.058)	0 (0)	3 (0.035)	5.8	0.5 to 50.3	0.087	39	1
* ADGRD1*	2 (0.20)^a^	46 (0.039)	5.2	0.6 to 19.9		0.061	42	0 (0)	2 (0.059)	2 (0.023)	8.6	0.6 to 119.3	0.056	34	1
* PLEKHA4*	2 (0.20)^a^	82 (0.069)	2.9	0.4 to 10.9		0.16	47	4 (0.078)	3 (0.088)	7 (0.082)	2.5	0.3 to 13.0	0.24	48	2
* DLGAP5*	1 (0.10)^a^	33 (0.028)	3.6	0.1 to 21.6		0.25	48	1 (0.019)	0 (0)	1 (0.012)	8.6	0.1 to 673.3	0.20	47	1

^a^Case numbers exclude non-HGSOC tumours present in original discovery cohort that were initially classified as HGS prior to obtaining tumour material.

^b^GnomAD 2.1.1 non-Finnish, non-cancer sub-population, *n* = 59,095.

^c^Fisher’s exact test results (*OR* odds ratio, *CI* confidence interval).

^d^*n* = 2572.

^e^Number of hemizygotes for chromosome X genes in MGRB unknown; % represents an estimate only.

^f^*n* = 1703.

^g^*n* = 4275.

^h^Extra HGSOC tumour from known *PALB2* germline LoF variant carrier (not from discovery cohort) included in total.

^i^Includes two tumours from pathogenic missense variant carriers (see text).

### Tumours from carriers of germline variants in proposed hereditary HGSOC genes

**Fig. 1:**
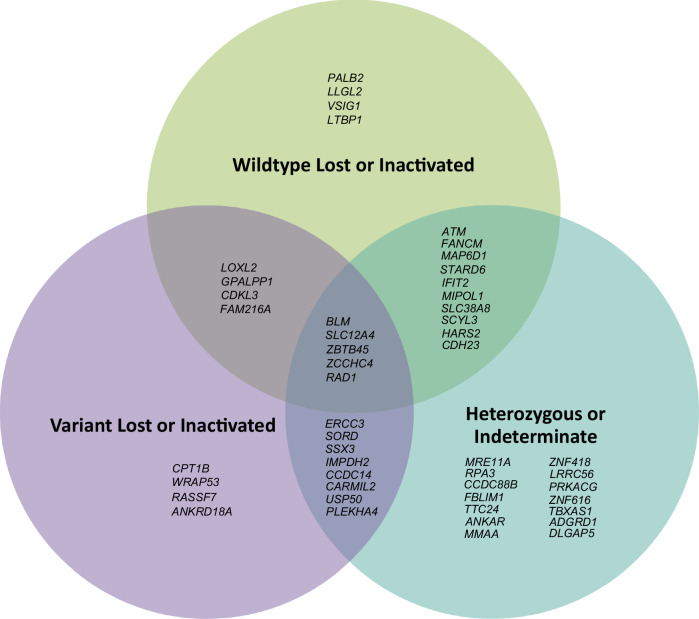
Venn diagram summarising tumour sequencing results for all genes of interest (proposed and candidate genes). Created using Microsoft PowerPoint.

For the 108 cases with WES data, mean sequencing depth across all target sequences was 90×, with 91% of bases on average covered to > 20×. Eighteen HGSOC tumours were from women carrying a germline LoF or known pathogenic missense variant in *PALB2*^4,5^ (*n* = 3), *ATM*^6^ (*n* = 5), *FANCM*^27^ (*n* = 2), *BLM*^28^ (*n* = 3), *MRE11A*^29^ (*n* = 2) or *ERCC3*^30^ (*n* = 3) (Table 2, Fig. 1). Biallelic inactivation through loss of the wildtype allele was observed in all three *PALB2* tumours. Three of five *ATM* tumours had biallelic inactivation, with one acquiring a predicted pathogenic somatic missense variant (CADD^31^ phred score = 26.6, REVEL^32^ score = 0.558) in a tumour with loss of the variant allele. For *BLM*, one of three tumours exhibited definite biallelic inactivation through loss of the wildtype allele; a second tumour had possible biallelic inactivation, with a heterozygous somatic stop-gain variant observed (phase unknown); the remaining tumour however showed loss of the variant allele. By contrast, *FANCM*, *MRE11A* and *ERCC3* tumours either remained heterozygous, or lost the variant allele. Promoter region bisulphite sequencing for four tumours with heterozygous results (one *ATM*, one *FANCM*, two *MRE11A*) showed no evidence of promoter hypermethylation. All three *PALB2* tumours with biallelic inactivation had calculated or estimated high HRD scores, whereas other tumours with biallelic inactivation showed no consistent pattern in this regard (Table 2). In summary, *PALB2* and *ATM* were the only proposed genes displaying consistent, verifiable somatic biallelic inactivation across multiple tumour samples from germline variant carriers.

**Table 2:** Tumour exome sequencing results for genes of interest (all proposed genes, and candidate genes with heterozygous and/or WT allele inactivation results only)

Gene of interest	Number of tumours	Sample ID	Age at diagnosis	HRD score	Germline transcript sequence variant and protein sequence change^c^	Overall tumour sequencing results	Biallelic inactivation present?
**Proposed genes**							
*PALB2*	3	PUB-WAF8U	54	High^b^	c.3113G>A p.(Trp1038Ter)	WT lost	YES
		PUB-RRQTJ	48	62	c.2325dupA p.(Phe776IlefsTer26)	WT lost	YES
		PUB-XXXXX^a^	48	68	c.2257C>T p.(Arg753Ter)	WT lost	YES
*MRE11A*	2	PUB-EVZW5	70	58	c.545-1G>T p.?	Heterozygous	NO
		PUB-ROW8I	57	High^b^	c.1726C>T p.(Arg576Ter)	Heterozygous	NO
*ATM*	5	PUB-2LRZJ	65	36	c.8307G>A p.(Trp2769Ter)	WT lost	YES
		PUB-H03DK	68	50	c.2135C>A p.(Ser712Ter)	WT lost	YES
		PUB-P2A20	63	61	c.8147T>C p.(Val2716Ala)	Variant lost with 2nd hit in WT allele^d^	YES
		PUB-GHWX3	44	High^b^	c.3756_3757dupTA p.(Lys1253IlefsTer4)	Heterozygous	NO
		PUB-BAD3Y	73	11	c.7271T>G p.(Val2424Gly)	Heterozygous	NO
*ERCC3*	3	PUB-1B2SB	60	34	c.1421dupA p.(Asp474GlufsTer2)	Variant lost	NO
		PUB-7BG9V	66	41	c.1762dupG p.(Glu588GlyfsTer16)	Variant lost	NO
		PUB-KBIYV	63	43	c.325C>T p.(Arg109Ter)	Heterozygous	NO
*BLM*	3	PUB-IBLSG	50	69	c.1933C>T p.(Gln645Ter)	WT lost	YES
		PUB-1G5DH	68	55	c.2206dupT p.(Tyr736LeufsTer5)	Heterozygous with 2nd hit (phase unknown)^e^	YES^f^
		PUB-0RH0G	65	Low^b^	c.2695C>T p.(Arg899Ter)	Variant lost	NO
*FANCM*	2	PUB-RTN4O	52	53	c.5791C>T p.(Arg1931Ter)	WT lost	YES
		PUB-Q6QAX	75	75		Heterozygous	NO
**Candidate genes**							
*MAP6D1*	3	PUB-R0JXJ	47	High^b^	c.493delG p.(Asp165ThrfsTer13)	Heterozygous with promoter methylation of one allele	NO^g^
		PUB-HLQJ5	82	35	c.266_294delGCGGACCGGGGGCGGGCGGCCGCAGGGGC p.(Arg89GlnfsTer86)	Variant lost with promoter methylation of WT allele	YES
		PUB-PRTO5	66	59		Heterozygous with promoter methylation of one allele	NO^g^
*RPA3*	2	PUB-MWTHR	58	33	c.118delA p.(Met40CysfsTer16)	Heterozygous	NO
		PUB-UGMNM	60	49	c.99+2T>C p.?	Heterozygous	NO
*STARD6*	2	PUB-XCS9D	61	36	c.545C>G p.(Ser182Ter)	Heterozygous	NO
		PUB-BGL6F	59	85	c.58_59delGA p.(Asp20TyrfsTer8)	WT lost	YES
*LLGL2*	3	PUB-C7OZT	78	40	c.2008delG p.(Ala670LeufsTer54)	Variant lost with promoter methylation of WT allele	YES
		PUB-EJ4NC	65	68	c.2869C>T p.(Arg957Ter)	WT lost	YES
		PUB-LKOD9	64	62		WT lost	YES
*CCDC88B*	3	PUB-U7P7F	43	31	c.898C>T p.(Gln300Ter)	Heterozygous	NO
		PUB-WF2F5	63	73	c.3834-1G>C p.?	Heterozygous	NO
		PUB-J3ODA	66	Low^b^	c.1028delT p.(Leu343ArgfsTer100)	Heterozygous	NO
*FBLIM1*	2	PUB-58A4L	63	73	c.1022_1031delACAGGGCTGG p.(Tyr341CysfsTer40)	Heterozygous	NO
		PUB-IXQKT	70	68	c.1078C>T p.(Arg360Ter)	Heterozygous	NO
*IFIT2*	3	PUB-9XXOO	42	81	c.325C>T p.(Arg109Ter)	WT lost	YES
		PUB-AF099	70	38		Heterozygous	NO
		PUB-SV5CQ	69	High^b^		Heterozygous	NO
*MIPOL1*	3	PUB-SUGLY	73	59	c.182C>G p.(Ser61Ter)	Heterozygous with promoter methylation of one allele	NO^g^
		PUB-NFUNH	61	High^b^	c.1192C>T p.(Arg398Ter)	Heterozygous with promoter methylation of WT allele	YES
		PUB-HLQJ5	82	35	c.1262+1delG p.?	Heterozygous with promoter methylation of WT allele	YES
*TTC24*	2	PUB-IFNQ0	65	39	c.1690delA p.(Ser564AlafsTer?)	Heterozygous	NO
		PUB-QY8W8	54	106	c.343C>T p.(Arg115Ter)	Heterozygous	NO
*SLC38A8*	2	PUB-SWU2E	65	High^b^	c.697G>T p.(Glu233Ter)	Heterozygous	NO
		PUB-CX65G	66	45		WT lost	YES
*ANKAR*	5	PUB-BFSYR	51	96	c.2853_2857delTAAAT p.(Lys952SerfsTer13)	Heterozygous	NO
		PUB-8U010	74	50	c.3059_3062delAGGA p.(Lys1020ThrfsTer22)	Heterozygous	NO
		PUB-5IGWB	49	51		Heterozygous	NO
		PUB-88NOV	59	58	c.3019delA p.(Met1007CysfsTer10)	Heterozygous	NO
		PUB-WANJC	56	48	c.3301-1G>A p.?	Heterozygous	NO
*SCYL3*	3	PUB-EVZW5	70	58	c.1474G>A p.?	Heterozygous with promoter methylation of WT allele	YES
		PUB-9SR2V	55	89	c.1444C>T p.(Arg482Ter)	WT lost	YES
		PUB-9ZUZK	68	47		Heterozygous	NO
*MMAA*	2	PUB-M4AJ2	65	32	c.439+4_439+7delAGTC p.?	Heterozygous	NO
		PUB-2TCR0	54	61	c.433C>T p.(Arg145Ter)	Heterozygous	NO
*ZNF418*	2	PUB-ATZ8R	53	76	c.1168C>T p.(Arg390Ter)	Heterozygous with promoter methylation of one allele	NO^g^
		PUB-YS9UF	58	High^b^	c.302_303delAG p.(Gln101ArgfsTer12)	Heterozygous with promoter methylation of one allele	NO^g^
*LRRC56*	1	PUB-K56S6	65	39	c.625-2A>C p.?	Heterozygous	NO
*HARS2*	2	PUB-IBW33	53	68	c.125C>G p.(Leu42Ter)	WT lost	YES
		PUB-FXXFL	62	23	c.324T>G p.(Tyr108Ter)	Heterozygous with promoter methylation of one allele	NO^g^
*PRKACG*	2	PUB-ZPSA2	60	47	c.-5_1dupCCGCCA p.(Phe1_?)	Heterozygous	NO
		PUB-SKYSL	70	86	c.19A>T p.(Lys7Ter)	Heterozygous	NO
*VSIG1*	1	PUB-9ZUZK	68	47	c.1211C>G p.(Ser404Ter)	WT lost	YES
*ZNF616*	3	PUB-A6JHW	41	29	c.610C>T p.(Gln204Ter)	Heterozygous	NO
		PUB-JOPWC	74	82	c.13-1G>A p.?	Heterozygous	NO
		PUB-0GFYL	65	66		Heterozygous	NO
*TBXAS1*	1	PUB-CX65G	66	45	c.240-1G>T p.?	Heterozygous	NO
*CDH23*	2	PUB-7IV8M	49	80	c.3006delC p.(Ser1003ProfsTer5)	Heterozygous with promoter methylation of one allele	NO^g^
		PUB-COP45	40	65	c.3109A>T p.(Lys1037Ter)	Heterozygous with promoter methylation of WT allele	YES
*LTBP1*	1	PUB-EOVJJ	65	70	c.864-1G>T p.?	Heterozygous with promoter methylation of WT allele	YES
*ADGRD1*	1	PUB-S3571	64	49	c.2T>C p.(Met1?)	Heterozygous	NO
*DLGAP5*	1	PUB-YU7VB	58	72	c.2112-1G>A p.?	Heterozygous	NO

Genes in each group are ordered according to gnomAD rank (see Table 1).

*WT* wildtype.

^a^Extra HGSOC tumour from known *PALB2* germline LoF variant carrier (not in discovery cohort).

^b^Quantitative HRD scoring not possible (see Methods); qualitative categorisation performed based on visual inspection of the tumour log_2_ CNV profile.

^c^Annotated to Ensembl canonical transcript and protein sequence (see Supplementary Table 5).

^d^ENST00000278616.4:c.2950C>A; ENSP00000278616.4:p.(Gln984Lys).

^e^ENST00000355112.3:c.1515G>A; ENSP00000347232.3:p.(Trp505Ter).

^f^Assuming somatic stop-gain variant is in WT allele.

^g^Based on additional bisulphite sequencing data showing presence of heterozygous gene promoter methylation in normal tissues and/or tumours (see Supplementary Table 4).

For full results (including other candidate genes with one or more tumours exhibiting variant allele loss), see Supplementary Table 3. Genes in each group are ordered according to gnomAD rank (see Table 1).

**Fig. 2:**
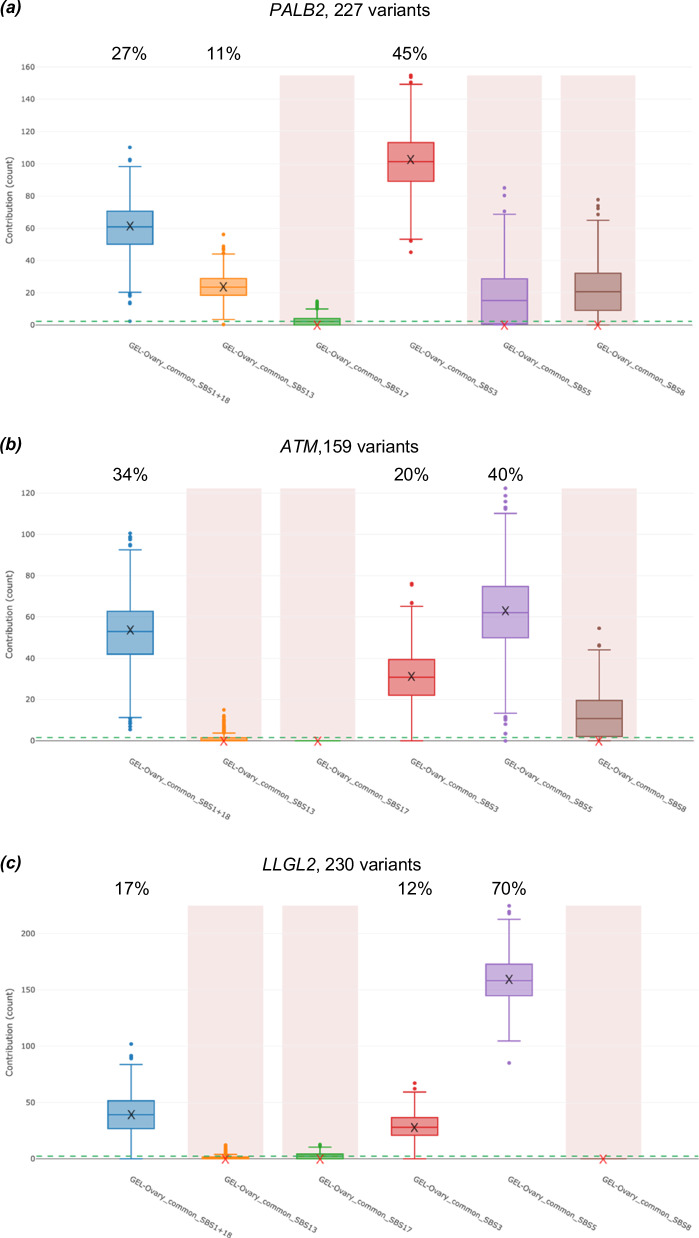
SIGNAL fitted mutational signatures (using the ovary-specific set and SIGNAL FitMS algorithm^33^) for pooled, unique WES somatic HGSOC tumour SBS mutations. **a** *PALB2* (*n* = 227), (**b**) *ATM* (*n* = 159) and (**c**) *LLGL2* (*n* = 230) inactivated tumours, with estimated percentage contributions. Signatures that did not pass the sparsity threshold and were not called are shaded. Crosses (‘X’) represent median contribution estimates; box plots provide the contribution estimate distribution for each signature (boxes and error bars denote mean +/- interquartile ranges and 95% confidence intervals, respectively); dashed lines represent sparsity filter threshold. Created using SIGNAL Analyse 2 platform^33^.

Mutational signature analysis using the SIGNAL Ovary signature set^33^ was performed for tumours with demonstrable loss or inactivation of the wildtype allele in multiple samples (three *PALB2* and *ATM* tumours each). As each tumour exome had a relatively small number of SBS somatic variants available for signature fitting (median 68 variants per tumour across all six samples), signature fitting was performed on pooled sets of unique SBS variants from each group of tumours sharing biallelic inactivation of the same gene of interest. The results (Fig. 2a, b) showed the *PALB2*-inactivated tumours to have a predominantly HR-deficient signature (GEL-Ovary_common_SBS3), whereas the *ATM*-inactivated tumours had a lesser HR-deficiency signature with others (i.e. GEL-Ovary_common_SBS5 and GEL-Ovary_common_SBS1+18) appearing more prominent.

### Tumours from carriers of germline variants in candidate familial HGSOC genes

Ninety-three tumours from carriers of germline LoF variants in one of the 43 candidate genes were available for sequencing (Supplementary Table 3). Nineteen genes (44%) demonstrated loss or promoter silencing of the wildtype allele in at least one tumour, with six genes (*SLC12A4*, *LOXL2*, *ZCCHC4*, *LLGL2, MIPOL1*, *SCYL3*) demonstrating this in multiple samples (Fig. 1). Except for *LLGL2*, none of the candidate genes demonstrated consistent inactivation of the wildtype allele in every available sequenced tumour from germline LoF variant carriers. Furthermore, nineteen genes (44%) demonstrated loss of the variant allele in at least one sample. Thirteen genes (30%) remained heterozygous in every sequenced tumour with no evidence of a somatic second hit (Fig. 1, Supplementary Table 3).

Bisulphite sequencing was successful in at least one tumour sample for 21 candidate genes displaying either heterozygosity or variant allele loss (Supplementary Table 4). Of these, thirteen genes showed no evidence of promoter methylation in 25 sequenced tumours. Eight genes (*CDH23*, *HARS2*, *LLGL2*, *LTBP1*, *MAP6D1*, *MIPOL1*, *SCYL3*, *ZNF418*) showed some degree of promoter methylation in at least one tumour, but only *MIPOL1* exhibited probable homozygous methylation in more than one sample. DNA from eight HGSOC tumours without germline or somatic variants in any of these eight genes additionally underwent promoter methylation analysis (Supplementary Table 4), to assess if epigenetic silencing might be limited to tumours from germline variant carriers requiring a second ‘hit’. All had promoter hypermethylation in at least one tumour, indicating this was not solely restricted to tumours with germline LoF variants.

Considering the bisulphite sequencing data together with the exome sequencing results, *LLGL2* emerged as the only candidate gene to show consistent biallelic inactivation in all sequenced tumours from germline LoF variant carriers (Table 2) in a manner analogous to the *PALB2* tumours, as one tumour with loss of the variant allele exhibited homozygous methylation of the promoter region for the remaining wildtype allele (Supplementary Fig. 2). Mutational signature analyses (Fig. 2c) showed the pooled somatic variants from the *LLGL2*-inactivated tumours were more like the *ATM*-inactivated tumours as opposed to the *PALB2*-inactivated tumours, with GEL-Ovary_common_SBS5 and GEL-Ovary_common_SBS1+18 signatures prominent.

### Additional investigation of tumours from *LLGL2* variant carriers

As the local control data affirmed a higher frequency of *LLGL2* germline LoF variants in HGSOC cases with all three tumours showing consistent biallelic inactivation, tumour IHC and WGS were performed to confirm loss of LLGL2 protein expression and any associated genomic features. Consistent with IHC data from The Human Protein Atlas (https://www.proteinatlas.org/ENSG00000073350-LLGL2/pathology/ovarian+cancer#ihc)^34–37^, all three control HGSOCs with an intact wildtype *LLGL2* allele from the exome sequencing data (Supplementary Fig. 3a) showed diffuse, strongly positive cytoplasmic staining. By contrast, all three *LLGL2* tumours with somatic loss or promoter silencing of the wildtype allele showed absent or weaker, patchier staining for the protein (Supplementary Fig. 3b), indicating loss of LLGL2 protein synthesis in at least a portion of tumour cells in these samples, with some residual non-specific background staining in stroma and other cells present. Mutational signatures derived from the WGS data (Supplementary Fig. 4) confirmed the signatures derived from the pooled WES data (Table 2, Fig. 2c), with no signatures uniquely associated with loss of *LLGL2* function other than SBS119, which was considered likely artefactual due to excess FFPE-associated T-to-C transitions in the mutational catalogues^38^. Calculated HRD scores for this data set were lower on average than characteristically seen in HGSOC tumours with significant HRD (mean 31 vs ≥ 63^39,40^); this was corroborated by the lower levels of HRD-associated signatures (particularly GEL-Ovary_common_SBS8) seen in the WGS mutational signatures (Supplementary Fig. 4).

## Discussion

Previously, germline exome sequencing of 510 likely hereditary HGSOC cases excluded for known genetic causes identified 43 genes with a higher frequency of LoF variants compared to non-cancer individuals in gnomAD. All 43 genes continue to show higher LoF variant frequencies in the cases when the analysis is repeated using 496 of these cases with confirmed HGSOC against a local control cohort, where 22 of the 43 genes show definite enrichment as illustrated by odds ratio confidence intervals exceeding one. While this data is reassuring in confirming the utility of gnomAD as a surrogate control cohort, the number of LoF variant carriers for each gene is small, and the observed associations are relatively modest with wide confidence intervals, making it difficult to interpret their actual significance.

In earlier work, we and others demonstrated the utility of integrating tumour sequencing data from germline variant carriers as a means of providing strong orthogonal evidence for or against the role of a new gene in cancer predisposition through identification of biallelic inactivation^9,16,41–43^. Here, we apply this approach to *PALB2* and *ATM*, which are genes known to predispose to breast cancer but where epidemiological evidence for an ovarian cancer association is equivocal (particularly for *ATM*)^4–6^. Biallelic inactivation, accompanied by the expected hallmarks of loss of homologous recombination repair function^44^, was observed in all HGSOC tumours from *PALB2* LoF variant carriers. In the *ATM* tumours, biallelic inactivation was observed in a majority of samples, although not universally, which is consistent with epidemiological data suggesting *ATM* has only a modest impact on ovarian cancer risk^6^. The ovarian tumours with *ATM* biallelic inactivation do not show any signatures related to HRD, which is atypical for HGSOC, but has been observed in breast tumours from *ATM* germline variant carriers exhibiting loss of the wildtype allele^16^. Together with the case-control data from elsewhere^4–6^, our results provide supportive evidence for *PALB2* and (to a lesser degree) *ATM* as moderate-risk HGSOC predisposition genes. Only *PALB2* though has a high enough lifetime HGSOC risk profile (~3 to 5%^4,5^) to consider risk-reducing surgery in certain individuals, as recommended for example by recent UK guidelines^45^.

The other proposed genes included in this study (*ERCC3, MRE11A, FANCM, BLM*) did not display this level of supportive evidence. *ERCC3* has been proposed as an ovarian cancer predisposition gene, based on a modest enrichment of LoF variants in ovarian cancer cases compared to gnomAD in a Spanish study^30^. Our tumour sequencing data from germline *ERCC3* LoF variant carriers shows no evidence of biallelic inactivation, with two out of three tumours furthermore displaying loss of the variant allele. This strongly argues against a role for *ERCC3* in ovarian cancer predisposition. The absence of biallelic inactivation in *MRE11A* tumours is consistent with recent studies that failed to demonstrate an association between *MRE11A* germline variants and ovarian cancer^46^, although it remains possible that the wildtype allele in these tumours may have been inactivated through an alternate genetic mechanism that was not detectable (e.g. deep intronic splicing variants). Only one of two *FANCM* tumours shows loss of the wildtype allele; coupled with the absence of any enrichment for LoF variants, it is unlikely that *FANCM* is a genuine HGSOC predisposition gene, notwithstanding the data from Dicks et al.^27^, which to date is the only study to suggest otherwise. Similarly, with loss of the variant allele observed in one out of three tumours, an association of *BLM* with HGSOC predisposition seems unlikely, especially given the weak epidemiological data^28,47^.

Exome sequencing results for the 43 novel candidate genes in general show no uniform biallelic inactivation, with only one gene- *LLGL2-* displaying biallelic inactivation in all sequenced tumours in the manner expected for a cancer predisposition gene (i.e. analogous to that seen for *PALB2* in this study). For many genes, the data for a role in HGSOC predisposition is equivocal since they either remain heterozygous or only a single tumour from multiple carriers exhibits biallelic inactivation. Furthermore, there is strong evidence that several of the candidate genes are unlikely to be genuine HGSOC predisposition genes (even when highly ranked for LoF variant enrichment in cases versus controls), since the variant allele is lost in one or more tumours (e.g. *WRAP53*). For some genes with ambiguous results, it is again possible that the wildtype allele is inactivated via an alternative mechanism, although promoter hypermethylation at least was excluded for many of them. While it is conceivable that some of the genes may act via an alternative pathway (e.g. haploinsufficiency^13^), convincing examples of such alternative mechanisms for hereditary cancer genes are uncommon, and their true extent and contribution remains uncertain. Overall, the tumour sequencing demonstrates the necessity of obtaining other orthogonal lines of evidence prior to making any firm assertions regarding the association of a novel gene with an increased cancer risk from exploratory case-control studies, due to the high possibility of false positive discoveries^48^.

Of the candidate genes, *LLGL2* has the strongest data supporting a role in HGSOC predisposition. The frequency of LoF variants in the cases remains higher when compared against the local controls (0.4% vs 0.035%) and all three available tumours from germline LoF variant carriers exhibit evidence of biallelic inactivation. Although no distinctive mutational signatures are associated with biallelic *LLGL2* loss, the HRD mutational signatures and scores are low in all three cases, which is less typical but not infrequent amongst HGSOC tumours^39,49^. Studies in Drosophila have found that *LLGL2* plays a role in asymmetric cell division, epithelial cell polarity and cell migration through interaction with atypical protein kinase C-containing complexes^50^. These complexes are also thought to interact with the homologous protein in mammalian and human epithelial cells to perform similar functions^51–53^. Its expression is known to be reduced in gastrointestinal tract malignancies^54–56^, with earlier work demonstrating a tumour suppressor role in *Drosophila* and zebrafish via its maintenance of correct cell polarity^57,58^. Recently, Gu et al.^59^ used bioinformatics analysis to demonstrate that low LLGL2 protein expression levels are significantly associated with higher epithelial ovarian cancer tumour grade and poorer survival; furthermore, they provided in vitro and in vivo functional data showing how LLGL2 acts to inhibit the migration and invasive abilities of ovarian cancer cells through regulation of cytoskeletal remodelling via interactions with ACTN1. This provides compelling evidence of a possible tumour suppressor role for *LLGL2* in pre-metastatic epithelial ovarian cancer cells, corroborating the germline and somatic genomic data presented here to support it as a potentially novel HGSOC predisposition gene.

Despite access to tumour material from germline LoF variant carriers, the study is limited by the small number of available samples per gene, reducing its power to validate any putative associations from the earlier discovery study. The use of WES also limited the degree of mutational signature analysis possible, owing to the relatively low number of somatic variants per tumour exome available for signature fitting. As highlighted before, the theoretical basis for this work relies on the assumption of a ‘two-hit’ mechanism for novel HGSOC predisposition genes, which may not necessarily be true for the candidate genes investigated here. Nonetheless, this has held true thus far for earlier well characterised HGSOC risk genes such as *BRCA1* and *BRCA2*.

In summary, this study provides corroborating evidence of a role for *PALB2* and *ATM* in HGSOC predisposition. Assuming novel HGSOC predisposition genes conform to a two-hit mechanism, many of the candidate genes identified previously can be excluded because they lose the variant allele in the tumour. A putative HGSOC predisposition role for *LLGL2* though is supported by the observation of consistent biallelic inactivation along with corroborating IHC, tumour genomic and case-control epidemiological data plus recent functional data indicating a possible tumour suppressor role in the relevant cell type^59^. This not only demonstrates the utility of incorporating analysis of larger case-control datasets with tumour sequencing in cancer predisposition gene research, but also highlights the need for larger cohorts of tumours from carriers of candidate gene variants for validating discoveries prior to translation for clinical use.

## Methods

### Case-control analyses using MGRB and Lifepool data

Cases comprised 496 women identified in the ViP cohort. These women were recruited from familial cancer centres in Australia and had a confirmed or suspected diagnosis of HGSOC and no pathogenic or likely pathogenic variant in a well-established ovarian carcinoma predisposition gene, as described previously^7^. The total number of ‘rare’ (gnomAD v2.1^8^ total AF ≤ 0.005) LoF variants within the GRCh37/hg19 Ensembl canonical transcript for each of the 43 candidate genes and six proposed (*PALB2*^4,5^, *ATM*^6^, *MRE11A*^29^, *FANCM*^27^, *BLM*^28^*, ERCC3*^30^) ovarian cancer predisposition genes were compared to the equivalent figures from MGRB^25^ (*n* = 2572) and Lifepool^26^ (*n* = 1703), separately and combined; this used the same filtering and ranking strategy as detailed before^7^. MGRB comprises elderly (> 75 years old), healthy individuals with no history of any major diseases (including cancer) with WGS data, from the ASPREE^60^ and 45 and Up^61^ cohort studies. Lifepool comprises Australian women recruited through their participation in population breast cancer screening, who at the time of blood collection had no known history of cancer; an unselected subset of these women (mean age 65 years, range 39 to 92 years) donated DNA to generate the WES data.

### Case selection, tumour sequencing, data processing and analysis

Formalin-fixed-paraffin-embedded (FFPE) HGSOC tumour blocks from 111 women (summarised in Supplementary Table 1) were obtained from diagnostic pathology laboratories. Each tumour was from a woman harbouring a germline LoF (stop-gain, frameshift or essential splice site) variant or known likely pathogenic or pathogenic missense variant (if categorised as such in ClinVar^62^) in one of the six accepted or proposed genes described above, or a LoF variant in one or more of 43 candidate genes from our earlier study^7^ (Table 1).

Tumour blocks were sectioned, slide-mounted and manually micro-dissected to collect tumour cells of purity ≥ 30% for DNA extraction, using the QIAamp DNA FFPE Tissue Kit as per the manufacturer’s instructions^63^ and as described previously^64^. Prior to sequencing, tumour DNA samples were re-quantified, and their quality assessed using the method described by van Beers et al. for FFPE-derived samples^65^, with minor modifications. Only samples with a van Beers polymerase chain reaction (PCR) result with at least one visible band at 100 bp were taken forward for exome sequencing.

DNA was sequenced using massively parallel sequencing for 108 of these samples- comprising WES with additional WGS for three samples in this set with biallelic inactivation of *LLGL2*- alongside Sanger sequencing for selected cases. The latter group included three other tumours (for *WRAP53* germline variant carriers) with no WES data, where Sanger sequencing targeting the variant of interest only was performed. WES libraries were prepared using 20 to 200 ng tumour DNA and one of the following library preparation protocols:

- Agilent SureSelect^XT HS^ Target Enrichment System^66^ with Agilent SureSelect Human All Exon v7 capture baits^67^ performed by us, followed by sequencing at AGRF (Australian Genome Research Facility, Melbourne, Australia) on the Illumina NovaSeq 6000 platform (150 bp paired-end reads).
- Vazyme VAHTS Universal Pro DNA Library Prep Kit^68^ and sequencing on the Illumina HiSeq 2500 platform (150 bp paired-end reads), both performed by GENEWIZ (Suzhou, China).
- Twist Bioscience Human Core Exome EF Multiplex Complete Kit^69,70^ and sequencing on the Illumina NovaSeq 6000 platform (150 bp paired-end reads), both performed by AGRF. Predominantly used for low-quality samples with only one 100 bp band on van Beers PCR.

For the three tumours with biallelic inactivation of *LLGL2*, WGS was performed on paired germline and tumour DNA (the latter extracted from FFPE samples as described above) by AGRF using the IDT xGen cfDNA & FFPE DNA Library Preparation Kit^71,72^ and Illumina NovaSeq 6000 platform (150 bp paired-end reads).

### Sequencing data processing and filtering

An in-house bioinformatics pipeline constructed using Seqliner v0.9.1^73^ was used to process raw tumour WES FASTQ data. Raw sequencing reads were quality checked using FastQC v0.11.6^74^, trimmed using cutadapt v2.1^75^ then aligned to the GRCh37/hg19 human reference genome using BWA-MEM v0.7.17^76^. Duplicate reads were filtered using Picard MarkDuplicates v1.119^77^ and metrics for sequencing coverage and depth calculated against the appropriate manufacturer’s bed alignment file for that exome library type. Base quality score recalibration and indel realignment were then performed on the filtered reads using GATK v3.8.0^78^.

Variants from the tumour exomes were called against a modified version of the appropriate manufacturer’s bed alignment file (where target regions were extended by 150 bp at either end), utilising pre-existing germline WES data for every sample^7^. Two separate annotated variant files per sample were generated: one with *all* tumour variants (including those present in the germline as well), referred to as the *tumour-only variant file*, using GATK HaplotypeCaller^79^; and another with somatic tumour variants *only* (i.e. excluding any present in the germline), referred to as the *somatic variant file*, using VarDict v1.4.6^80^, GATK MuTect2^81^ and VarScan v2.3.7^82^. For one tumour sample without paired germline exome data (PUB-XXXXX from a *PALB2* variant carrier), somatic variants were called and output to a vcf file using VarScan v2.3.7, Platypus 0.8.1^83^ and GATK UnifiedGenotyper^84^. All variants in the vcf files were subsequently annotated for predicted consequences using Ensembl VEP database v85^85^ and LoFTEE v1.0.3^86^.

Somatic variant files were filtered (Supplementary Fig. 1a) using a custom script in R (v4.0.2 (2020) with tidyverse v1.3.0 installed) to remove all low-quality somatic variants that were likely sequencing artefacts as well as any present in gnomAD at AF > 0.0001, retaining variants within the exons and splice regions targeted by the exome capture. This filtering for somatic variants incorporated an adjustment of the variant allele frequencies (VAF) cut-off based on the estimated tumour purity for each case (see below). With regards to sample PUB-XXXXX, somatic variants were identified through more stringent filtering (Supplementary Fig. 1a) to ensure all possible germline variants as well as sequencing artefacts were removed.

For the WGS data, a pipeline deployed by AGRF was used, in which sequence reads were checked against internal quality control measures and screened for sequence contamination, prior to alignment and duplicate marking using Illumina’s DRAGEN Bio-IT platform v3.10.8 (v07.021.624.3.10.8) and the GRCh38/hg38 human reference genome. Somatic variant calling was subsequently undertaken using GATK MuTect2 (v4.1.7.0), followed by basic variant filtering for likely germline, low quality, contamination, orientation bias and sequence artefacts; remaining variants were annotated using Ensembl VEP database v105. Somatic variant files were then filtered in an equivalent manner to the tumour exome somatic variant files, with some minor differences (Supplementary Fig. 1b).

### Tumour copy number variant (CNV) analysis

On- and off-target tumour WES reads were used to generate genome-wide CNV and BAF data with CNVkit^87^ and PureCN^88^, normalised against process-matched germline DNA samples from the same sequencing batch. CNVkit bin sizes were set for the Twist exomes using the ‘autobin’ function (on-target bins ~1600 bp, off-target bins ~60 kbp), and standardised on- and off-target bins of 267 bp and 50 kb respectively were used for the remaining Agilent SureSelect v6/v7 exomes. PureCN utilised default bins of ~400 bp and ~50 kbp for on-target and off-target regions respectively in all exomes, regardless of the library platform. PureCN data was visualised and tabulated using the package’s standard output functions^89^, whereas CNVkit data for CNVs (log_2_ ratios, normalised per bin) and BAF values (incorporating manual filtering to retain data points at the extremities) were visualised and tabulated in NEXUS v.9.0 software using the default settings^90^ or directly viewed using the CNVkit output.

### Purity assessment

Tumour purity was estimated for the purpose of somatic variant filtering and determining LoH for the genes of interest based on a mean average of the following:

- PureCN estimate, derived from the closest fitting solution selected following visual inspection of all solutions produced by the algorithm for a given sample (usually but not always one of the top three ranked solutions). Estimated ploidy and other data parameters for the selected solution were checked against the corresponding CNVkit results for that sample to ensure the most appropriate solution was picked.
- Visual estimate from the CNVkit plot for that sample (using the calculated log_2_ ratios), based on the relative heights of different CN gains and losses versus the baseline.
- Estimate from the read frequency of selected somatic variants (primarily in *TP53*), where present in conjunction with CN loss of the wildtype allele.

### Homologous recombination deficiency (HRD) scoring

HRD scores (comprising the sum of telomeric allelic imbalances, large-scale state transitions (LST) and loss of heterozygosity across the tumour genome, based on the method initially outlined by Timms et al.^91^ and subsequently modified by Telli et al.^40^ for array-based data) were calculated using the PureCN output for WES data, utilising the same solution as that used for the tumour purity estimate. This was achieved with a custom R script (adapted from one originally used by Marquard et al.^92^ for ASCAT output^93^) to estimate and sum unweighted scores from the chosen solution for LoH^94^, telomeric allelic imbalance (TAI)^95^ and LST^96^. Calculated LST scores (and thus overall HRD scores) were not adjusted for ploidy (as proposed by Timms et al. to account for falsely elevated HRD scores with increasing ploidy in both HR intact and deficient samples^91^), due to the variability of the estimated ploidy between the different PureCN solutions for each sample and resulting uncertainty as to the true ploidy value. For the three tumour samples with WGS data, FACETS^97^ was used to generate genome-wide CNV and BAF results (cval 1500, clonal events only) for calculation of HRD scores, again using the custom R script.

For all samples, an HRD score of ≥ 63 indicates significant HRD in HGSOC, as recommended elsewhere^39^. Quantitative HRD scoring could not be performed using this method for fifteen tumours with lower-quality WES data (i.e. due to degraded FFPE material or low tumour purity) and an excessive number of PureCN solutions (> 10). Instead, HRD was qualitatively estimated as ‘Low’ or ‘High’, based on visual inspection of the CNVkit tumour log_2_ CNV profiles (data not shown).

### Variant allelic status determination

For each tumour, the exome sequencing tumour-only and somatic variant files were jointly interrogated using the tumour purity-adjusted VAF to assess the allelic status of the variant of interest and/or to identify any additional somatic LoF or predicted pathogenic missense variants. Sequencing reads across variants of interest were manually reviewed in IGV^98^. Copy number variant (CNV) data for each tumour (see above) was additionally interrogated at each locus to corroborate the determination of allelic status from the VAF results. Sanger sequencing (as described below) was performed for any tumour sample where the allelic status for the gene of interest remained uncertain or ambiguous or where WES data was unavailable (i.e. for three *WRAP53* tumours). For those tumours with sufficient DNA where there was no evidence of allelic loss or inactivating somatic point variants involving the gene of interest, bisulphite sequencing of their associated promoter CpG islands (if present) was performed as described below to assess for promoter hypermethylation.

### Mutational signature analyses

Mutational signatures were calculated from pooled unique tumour single base substitution (SBS) somatic variants using the SIGNAL FitMS algorithm and website Analyse 2 platform^99^, fitting against the ovary-specific SBS signature set (bootstraps = 1000, sparsity threshold = 1%, *p*-value = 0.05)^33^. For the three *LLGL2* tumours with WGS data, an additional algorithm (FFPEsig) was applied post-filtering to the mutational catalogues to remove excess C-to-T transition artefacts introduced during the FFPE preservation process^38^, prior to signature fitting on individual samples (bootstraps = 100, dynamic sparsity threshold with sparsity scaling factor = 10, *p*-value = 0.05).

### Tumour sanger and bisulphite sequencing

Primers targeting an amplicon containing the variant of interest were designed using Primer3^100–102^ (Supplementary Table 2) and checked using the UCSC in silico polymerase chain reaction (PCR) tool^103^. An M13 sequence (GTAAAACGACGGCCAGT) was added to the forward primer if the amplicon sequence was < 200 bp, and/or the variant was close to the primer binding site. Primers were optimised with female reference DNA using a standard touchdown PCR program and reaction mixture at different Mg concentrations (1.5 mM or 4 mM), or using a gradient temperature PCR if required, to determine the optimal annealing temperature and PCR conditions.

Tumour DNA samples selected for bisulphite sequencing were subjected to bisulphite conversion using the EpiTect Fast Bisulphite Conversion kit according to the manufacturer’s protocol^104^. Bisulphite sequencing primers targeting the 5′-promoter region CpG islands for genes of interest were designed using MethPrimer^105^ (Supplementary Table 2) and checked using the Bi-Search ePCR tool^106,107^, with M13 sequences added to selected forward primers if initial optimisation failed. Primers were optimised using a gradient temperature PCR and reaction mixture at different Mg concentrations (1.5 mM, 2.75 mM, or 4 mM) using normal and bisulphite-treated female reference DNA, to determine the optimal annealing temperature and PCR conditions.

Once optimum conditions were established for the relevant primer pairs, PCR followed by BDT Sanger sequencing was performed on 10 to 20 ng tumour DNA (untreated or bisulphite-treated, as required) using standard methods. Sequencing chromatograms were visualised in Geneious 8.1.9^108^.

### Tumour immunohistochemistry (IHC)

IHC was performed using an Abnova primary monoclonal antibody (M06, clone 4G2) against the N-terminal end of the LLGL2 protein (amino acids 101 to 199) at 1:200 dilution in conjunction with an Agilent Dako anti-mouse secondary antibody conjugated to horseradish peroxidase. Unless otherwise specified, all reactions and washes were performed using standard TBS/T buffer. Four-micron tumour sections mounted on Superfrost-coated slides were obtained from the three HGSOC tumours with *LLGL2* biallelic inactivation, along with three positive control HGSOC tumours with germline wildtype *LLGL2* alleles and no evidence of somatic biallelic loss involving this locus. All sections were first dewaxed using xylene and dehydrated using 100% and 70% ethanol washes. Antigen-retrieval was then performed in citric acid buffer (pH 6) at high pressure for three minutes, followed by blocking of endogenous peroxidases using 3% hydrogen peroxide for fifteen minutes at room temperature. Blocking of endogenous staining was achieved using Agilent Dako 5% normal goat serum for 45 min at room temperature, and either Abnova primary monoclonal mouse antibody to LLGL2 at 1:200 dilution or additional goat serum (negative antibody control sections only) added prior to overnight incubation at 4 °C. Sections were subsequently incubated with Agilent Dako anti-mouse secondary antibody (1:200 dilution) conjugated to horseradish peroxidase for 45 min at room temperature, then stained with DAB (diluted in Agilant Dako substrate buffer) for five minutes followed by counter-staining using haematoxylin and Scott’s tap water. Finally, sections were cover-slipped, reviewed under 40X magnification, and photographed using an Olympus VS120 slide-scanning microscope. Full details of steps, volumes and reagents used can be found in the provided reference^109^.

## Supplementary information

Supplementary Materials

## Data Availability

Germline exome sequencing data referenced in this study^7^ has been previously deposited in the European Genome-phenome Archive (https://ega-archive.org/) under the study ID EGAS00001004235 and dataset accession code EGAD00001006030. Tumour exome and genome VCF datasets generated and analysed during the current study are available in the same repository under the study ID EGAS50000000770 and dataset accession codes EGAD50000001131 and EGAD50000001132. These datasets are available upon request on application to the linked Data Access Committee at dac@petermac.org (https://www.ebi.ac.uk/ega/dacs/EGAC00001003451). All other data used or analysed for this study are either available within the article and its supplementary information files, or from the corresponding author upon reasonable request. Other referenced datasets are accessible from gnomAD (http://gnomad.broadinstitute.org)^8^, MGRB (https://sgc.garvan.org.au/)^25^, The Human Protein Atlas (https://www.proteinatlas.org/)^35^ and ENCODE (https://www.encodeproject.org)^110^.

## Code availability

R script used for data analysis available at https://rpubs.com/deepsubs/npj_genom_med_2024. The Seqliner code is separately available at http://bioinformatics.petermac.org/seqliner/. All other publicly available code used during exome sequence data processing and variant calling are available via the provided links in the references.
