# Supplementary material for: Assessment of candidate high-grade serous ovarian carcinoma predisposition genes through integrated germline and tumour sequencing
Source: NPJ Genom Med. 2025 Jan 10;10:1. doi: 10.1038/s41525-024-00447-3 (PMC11724014; doi:10.1038/s41525-024-00447-3)
Supplement: Supplementary file 1 — Supplementary Materials [file 41525_2024_447_MOESM1_ESM.pdf]

Supplementary Materials

Supplementary Tables

Supplementary Table 1

Summary of patient cohort characteristics for all sequenced tumours.

	**Number (%)**
**Total patients**	111 (100)
**Age at diagnosis of ovarian carcinoma**	
40-49	18 (16)
50-59	26 (23)
60-69	44 (40)
70-79	18 (16)
≥ 80	5 (5)
**Personal history of cancer**	
Breast (excl. DCIS)	11 (10)
Other (incl. breast DCIS)	10 (9)
No history of cancer	90 (81)
**Family history of ovarian or breast cancer (1st and 2nd degree relatives only)**	
One ovarian cancer case (no breast cancer)	10 (9)
One breast cancer case (no ovarian cancer)	27 (24)
≥ 2 breast cancer cases (no ovarian cancer)	10 (9)
≥ 2 ovarian and breast cancer cases	8 (7)
No known cases of breast or ovarian cancer	56 (50)

Supplementary Table 2

List of primer targets and sequences used. PCR reaction conditions are available from the authors on reasonable request.

**Gene**	**Targeted Variant/ Sequence[%]**	**Bisulphite-Converted DNA?**	**Sequence (5`-3`)**	**Product Size (excl. M13 sequence)**
ANKAR	CpG Island	YES	GTAAAACGACGGCCAGTGGAGTAGTTTAGTTATTTTAGGGTT*	135 bp
			CTAAAAAAAACTTTCAAAACTTTTC	
ANKRD18A	CpG Island	YES	TTTTGATGGTAGTTTTGTGGATTT	137 bp
			CACCATAAAAAAACTCTTCAACTTC	
ATM	*CpG Island*	*YES*	GTAAAACGACGGCCAGTAAAGGTTTTTTTGTTTAGTATAGT*	*109 bp*
			TAATTTTAATCATATTATTACCCTC	
CARMIL2	CpG Island	YES	GTTTGGGTTTTAGGTTTTTTGTGT	182 bp
			TTTTAAAACTACACTACTTCCTCCC	
CCDC14	CpG Island	YES	GTAAAACGACGGCCAGTTTTGTAGGTGATTGAAGTTAGGAGAG*	181 bp
			AAATACAAAAAACACCTAATCTTCCC	
CCDC88B	CpG Island	YES	GGTTGGTTGTTTTTAGTTTTATAT	144 bp
			CCTCAACTCCTCCTACAAAC	
CDH23	CpG Island	YES	GGGGATTTATATATTTTTTTAAATT	149 bp
			ATACCAACTCCTAAAAACAACCAAC	
CDKL3	CpG Island	YES	TTTATTAGGTTTAAGGTTTTAAAAA	147 bp
			AAACCACAAAATAAACTAAAAC	
CPT1B	CpG Island	YES	GTAAAACGACGGCCAGTTTGAAAGTTTTAGGAGTTTTAAGGA*	124 bp
			AACCCTACCTCTATCCAAACC	
DLGAP5	CpG Island	YES	TGAAATTTTAATTAAATAAAAAGTAAAGAA	140 bp
			AATTAAAACCCCTCCAATCAAC	
FAM216A	CpG Island	YES	GTAAAACGACGGCCAGTTTTAAGTTTGGATATTTGAGTAATAA*	112 bp
			AAAATCACCATACAAATCAAAC	
FANCM	CpG Island	YES	TGGATTTATTTTATTAATTGTTTAGTG	181 bp
			TTAAAACCATAAAAACCACCTTTC	
FBLIM1	CpG Island	YES	TTTTAGTTTTTTGGTTTTTTTT	300 bp
			CTCCCCTTACCTAACCCTAACTAAA	
GPALPP1	c.540+1G>A	NO	GTAAAACGACGGCCAGTAAACTGACCAAAGGAGATGA*	199 bp
			AGACGTTCATCCAATTTGTT	
	CpG Island	YES	GTAAAACGACGGCCAGTTAAGGTTTAGTTAATTATAGGTTTTA*	128 bp
			TAATCACAAATATAAATCTATCCAC	

HARS2	CpG Island	YES	GTAAAACGACGGCCAGTGAAGTTGGTTATTAAGGGAATTTG*<br>CAAACACCAAAAAAAATAAAAAACAC	132 bp
IFIT2	c.325C>T	NO	GTAAAACGACGGCCAGTCTGGTCACCTGGGGAAACTA*<br>ACTTTAACCGTGTCCACCCT	166 bp
IMPDH2	CpG Island	YES	TTTTAGATGTTTTAAAGTGAGTTT<br>CAACACAACAACTCTTCAACTAC	200 bp
LLGL2	c.2008delG	NO	GTAAAACGACGGCCAGTGCGTCAAGTCCCTCAAGAAG*<br>CTCTGAGCCGAACCTGGG	154 bp
	CpG Island	YES	GTAAAACGACGGCCAGTTAGGAGAGTAGGAGAATAGGTGTTT*<br>AAATAACAAAAACAAAAACTAAACC	191 bp
LOXL2	c.1880+1G>A	NO	GTAAAACGACGGCCAGTCTCCCATCTGAGGCCTGC*<br>TCCTCCCAGATCCACAACAA	150 bp
	CpG Island	YES	GTTTTTATTTGGTTTTAGGGATT<br>TTAATACTTACTTTCTCCAACCATC	148 bp
LRRC56	CpG Island	YES	TTAGGTTGAGTTTTTAGGTGAGAGT<br>TAAAAACAAAAATCCTCAAAAAACC	187 bp
LTBP1	CpG Island	YES	GTAAAACGACGGCCAGTGTATGGGGTTTTTGAAAGGATAG*<br>ACCCTCCACATCCACTAAAATTAC	135 bp
MAP6D1	CpG Island	YES	GTAAAACGACGGCCAGTTTTTTTAGTGTTATTTTATAAATGGTTT*<br>ACAACCTAACACAAATACAACAATC	142 bp
MIPOL1	c.182C>G	NO	GTAAAACGACGGCCAGTTCTGAGAAAAGTATGCATCGGA*<br>AGTGCAGTAAACAGACTCATCA	138 bp
	CpG Island	YES	GAGTTAGGAGGTTTTGAGTTAGTGG<br>AACTAATCTATACCCACCACTATCATTCT	178 bp
MRE11A	CpG Island	YES	TTTGGGAATTTGTAGAAATAGGAGA<br>AATTCTCAAAAACAAATTCAAACC	164 bp
RAD1	c.168_172delAAAGT	NO	TTTGTCTACTGAAACCTTCCGA<br>CCAGCCTTGACAACGTTAGG	225 bp
	c.325C>T	NO	CCAATTCAGAAAATGCTTCACGG<br>TTCTCTACACACAACTGTCATCA	379 bp
	CpG Island	YES	GAGTTTTAGATAATTTTATTTATTAGTT<br>AAACTAACTTCACTTCCTCC	185 bp
RASSF7	*CpG Island*	*YES*	*TTGAGTAGTTAATAGGGGTGTTAGG*<br>*AAAAACTCTCCCCAATTAAAATC*	*226 bp*
RPA3	CpG Island	YES	ATTTTTAAAAAGTTTTTTGTGTAAAATAAA<br>CAATCATAAACTCCCAAATCC	132 bp
SLC12A4	c.3G>A	NO	CCCCTCGAGGTTGTCATAGT<br>CGGAGGAAGTGAGCGCAG	194 bp
	CpG Island	YES	ATATTTTGGTTAGAGTGTAGGTAGG<br>AACTCCTCCCCTATAAACCCC	266 bp
STARD6	c.58_59delGA	NO	GTAAAACGACGGCCAGTTTTAACCACTTTCCAGCCTGA*<br>GCAGATAGATGGACTTCAAGGC	92 bp
	CpG Island	YES	GAATTTATTTGGAAAAATTGGAGTT<br>AAAACTTACTAATCTCCCTTCAACC	140 bp
SCYL3	CpG Island	YES	GTAAAACGACGGCCAGTATAGAAAGTATTATGTTAAGATAGGAGTTA*<br>AAAAATATAAAAAATAAAAAACCAAC	250 bp
TBXAS1	CpG Island	YES	GTAAAACGACGGCCAGTATTTGTTATTTTGAGTTTGTGTTTT*<br>CAACCATACTAAACTAAACCCC	300 bp
WRAP53	c.1225C>T	NO	GTAAAACGACGGCCAGTGATGCTGAGCTCCTGTGC*<br>CACGGGTCCAGATCGAAGTA	107 bp
	c.682delG	NO	GTAAAACGACGGCCAGTCCTTTTCCCTCCGAGTGACT*<br>GGCTGGGCTGAGGACATC	122 bp
	c.904_905insT	NO	GTAAAACGACGGCCAGTCTTCTCCCCGGATGGCTC*<br>TCCCTCTCCTCCTCCCTTG	140 bp
	c.17_18delCT	NO	GTAAAACGACGGCCAGTAGGCTAATCTCCGCTGTGC*<br>GGAGAAGGATGGGCTGGAG	111 bp
	CpG Island	YES	GGAGTTTTAGGGTTTGATGGG<br>TTAAAAACACTATATTCCTTAACACC	185 bp
ZBTB45	CpG Island	YES	GTATTTGAAAGGATTTTAATTTGTT<br>ACAAACCTTTATTCTAAAATATCCAAAC	161 bp
ZCCHC4	CpG Island	YES	GTAAAACGACGGCCAGTGGTTTTTAGGAATGGGTTTGAA*<br>AATCCAAAAAAAACACCACCTC	92 bp
ZNF418	CpG Island	YES	GATTTGAGGGTAGGGAAGGTATAAT<br>AAACCCAAAAAATAAAAAATCCTTCT	162 bp

*Includes M13 sequence (GTAAAACGACGGCCAGT).

[%]Annotated to Ensembl canonical transcript (see Supplementary Table 5).

Supplementary Table 3
Tumour exome sequencing results for all genes of interest (proposed and candidate gene), including those with no WT allele inactivation. Genes in each group are ordered according to gnomAD rank (see Table 1).

Gene of Interest	Number of Tumours	Sample ID	Age at Diagnosis	HRD Score	Germline Transcript Sequence Variant and Protein Sequence Change[%]	Exome Sequencing Results	CNV Status	Bisulphite Sequencing Results	Overall Tumour Sequencing Results	Biallelic Inactivation Present?
					**PROPOSED GENES**					
PALB2	3	PUB-WAF8U	54	High*	c.3113G>A p.(Trp1038Ter)	WT lost	Allelic imbalance, CN loss	Not performed	WT lost	YES
		PUB-RRQTJ	48	62	c.2325dupA p.(Phe776IlefsTer26)	WT lost	Allelic imbalance, CN loss	Not performed	WT lost	YES
		PUB-XXXXX[#]	48	68	c.2257C>T p.(Arg753Ter)	WT lost	Allelic imbalance	Not performed	WT lost	YES
MRE11A	2	PUB-EVZW5	70	58	c.545-1G>T p.?	Heterozygous	CN neutral	Unmethylated	Heterozygous	NO
		PUB-ROW8I	57	High*	c.1726C>T p.(Arg576Ter)	Heterozygous	CN neutral	Unmethylated	Heterozygous	NO
ATM	5	PUB-2LRZJ	65	36	c.8307G>A p.(Trp2769Ter)	WT lost	Allelic imbalance	Not performed	WT lost	YES
		PUB-H03DK	68	50	c.2135C>A p.(Ser712Ter)	WT lost	Allelic imbalance	Unmethylated	WT lost	YES
		PUB-P2A20	63	61	c.8147T>C p.(Val2716Ala)	Variant lost with 2nd hit in WT allele[a]	Allelic imbalance	Not performed	Variant lost with 2nd hit in WT allele[a]	YES
		PUB-GHWX3	44	High*	c.3756_3757dupTA p.(Lys1253IlefsTer4)	Heterozygous	CN neutral	Unmethylated	Heterozygous	NO
		PUB-BAD3Y	73	11	c.7271T>G p.(Val2424Gly)	Heterozygous	CN neutral	Not performed	Heterozygous	NO
ERCC3	3	PUB-1B2SB	60	34	c.1421dupA p.(Asp474GlufsTer2)	Variant lost	Allelic imbalance	Not performed	Variant lost	NO
		PUB-7BG9V	66	41	c.1762dupG p.(Glu588GlyfsTer16)	Variant lost	Allelic imbalance	Not performed	Variant lost	NO
		PUB-KBIYV	63	43	c.325C>T p.(Arg109Ter)	Heterozygous	Allelic imbalance, CN gain	Not performed	Heterozygous^ with amplification of variant allele	NO
BLM	3	PUB-IBLSG	50	69	c.1933C>T p.(Gln645Ter)	WT lost	Allelic imbalance	Not performed	WT lost	YES
		PUB-1G5DH	68	55	c.2206dupT p.(Tyr736LeufsTer5)	Heterozygous with 2nd hit (phase unknown)[b]	CN loss	Not performed	Heterozygous with 2nd hit (phase unknown)[b]	YES[@]
		PUB-0RH0G	65	Low*	c.2695C>T p.(Arg899Ter)	Variant lost	CN loss	Not performed	Variant lost	NO

FANCM	2	PUB-RTN4O	52	53	c.5791C>T p.(Arg1931Ter)	WT lost	LoH	Not performed	WT lost	YES
		PUB-Q6QAX	75	75		Heterozygous	CN neutral	Unmethylated	Heterozygous	NO
					**CANDIDATE GENES**					
MAP6D1	3	PUB-R0JXJ	47	High*	c.493delG p.(Asp165ThrfsTer13)	WT lost	CN gain	Heterozygous methylation	Heterozygous with amplification of variant allele and promoter methylation of one allele (unknown which allele is methylated)	NO[$]
		PUB-HLQJ5	82	35	c.266_294delGCGGAC CGGGGGCGGGCGGC CGCAGGGGC p.(Arg89GlnfsTer86)	Variant lost	Allelic imbalance	Homozygous methylation	Variant lost with promoter methylation of WT allele	YES
		PUB-PRTO5	66	59		Variant lost	CN gain	Heterozygous methylation	Heterozygous with amplification of WT allele and promoter methylation of one allele (unknown which allele is methylated)	NO[$]
SLC12A4	5	PUB-5UQVG	66	90	c.1109dupT p.(Gly372ArgfsTer20)	Variant lost	CN loss, LoH	Not performed	Variant lost	NO
		PUB-5L8T4	72	42		Heterozygous	CN loss	Not performed	Heterozygous	NO
		PUB-KH12P	71	42	c.3G>A p.(Met1?)	WT lost	CN loss, LoH	PCR failed	WT lost^	YES
		PUB-SWU2E	65	High*		Heterozygous	CN loss	PCR failed	Heterozygous^	NO
		PUB-YS9UF	58	High*		No coverage	Allelic imbalance, CN loss	PCR failed	WT lost^	YES
SORD	4	PUB-PPGJ2	70	High*	c.757delG p.(Ala253GlnTer27)	Heterozygous	CN neutral	Not performed	Heterozygous	NO
		PUB-WJLZJ	54	50		Heterozygous	Allelic imbalance	Not performed	Heterozygous	NO
		PUB-A6JHW	41	29		Variant lost	Allelic imbalance	Not performed	Variant lost	NO
		PUB-K3JI1	73	81		Heterozygous	CN gain	Not performed	Heterozygous	NO
CPT1B	2	PUB-8SDCZ	68	41	c.562-2A>G p.?	Variant lost	Homozygous copy loss	Not performed	Variant lost	NO
		PUB-L09J6	76	45	c.1459-1G>C p.?	Variant lost	Allelic imbalance, CN loss	Sanger failed	Variant lost	NO
ZBTB45	4	PUB-7V374	65	42	c.616C>T p.(Arg206Ter)	Heterozygous	CN gain	Unmethylated? (noisy chromatogram)	Heterozygous	NO
		PUB-R6ZS2	52	91	c.416_417delCT p.(Pro139ArgfsTer33)	Variant lost	CN loss	Not performed	Variant lost	NO

		PUB-WIZBT	60	85	c.833C>G p.(Ser278Ter)	WT lost	Allelic imbalance, high copy gain	Not performed	WT lost with amplification of variant allele	YES
		PUB-BT3YN	61	40	c.734dupT p.(Thr246HisfsTer5)	Heterozygous	CN neutral	Unmethylated? (noisy chromatogram)	Heterozygous	NO
LOXL2	3	PUB-TV5KL	69	45	c.1880+1G>A p.?	WT lost	CN loss	Not performed	WT lost^	YES
		PUB-LYFY5	62	69		WT lost	Allelic imbalance, CN loss	Unmethylated	WT lost^	YES
		PUB-PWBKB	70	74		Variant lost	CN loss, LoH	Unmethylated	Variant lost^	NO
SSX3	2	PUB-9XXOO	42	81	c.*4+1G>A p.?	Heterozygous	CN neutral	No CpG island	Heterozygous	NO
		PUB-TI67L	57	85		Variant lost	Allelic imbalance, CN loss	No CpG island	Variant lost	NO
ZCCHC4	10	PUB-TENNY	82	25	c.558_559insAT p.(Thr246HisfsTer5)	Variant lost	Allelic imbalance, CN loss	Sanger failed	Variant lost	NO
		PUB-SUGLY	73	59	c.1286delA p.(Asn429IlefsTer54)	WT lost	Allelic imbalance, CN loss	Not performed	WT lost	YES
		PUB-FWG1Q	41	71		Heterozygous	CN neutral	Sanger failed	Heterozygous	NO
		PUB-XCS9D	61	36		Variant lost	Allelic imbalance	Sanger failed	Variant lost	NO
		PUB-ZRDAA	63	98		Heterozygous	Allelic imbalance, CN loss	Sanger failed	Heterozygous	NO
		PUB-EZ87I	55	41		Heterozygous	CN neutral	Sanger failed	Heterozygous	NO
		PUB-S3571	64	49		Heterozygous	Allelic imbalance	Not performed	Heterozygous	NO
		PUB-H03DK	68	50	c.1522delC p.(His508IlefsTer14)	Heterozygous	CN loss	Not performed	Heterozygous	NO
		PUB-78V9C	42	63		Heterozygous	CN loss	Not performed	Heterozygous	NO
		PUB-7GYYG	58	49		WT lost	CN gain	Sanger failed	WT lost	YES
RPA3	2	PUB-MWTHR	58	33	c.118delA p.(Met40CysfsTer16)	Heterozygous	CN neutral	PCR failed	Heterozygous	NO
		PUB-UGMNM	60	49	c.99+2T>C p.?	Heterozygous	Allelic imbalance	PCR failed	Heterozygous	NO
IMPDH2	3	PUB-W97N2	45	High*	c.12C>A p.(Tyr4Ter)	Variant lost	Allelic imbalance, CN loss	Not performed	Variant lost	NO
		PUB-WF2F5	63	73	c.278_290dupACAACT GTACACC p.(Glu98GlnfsTer5)	Heterozygous	Allelic imbalance	Unmethylated? (noisy chromatogram)	Heterozygous	NO
		PUB-QJ5BB	83	54	c.1061_1064dupCACG p.(Arg356ThrfsTer10)	Heterozygous	Allelic imbalance, CN gain	Not performed	Heterozygous	NO

GPALPP1	3	PUB-25VQV	63	91	c.229_230delAG p.(Arg77GlufsTer5)	Variant lost	Allelic imbalance, CN loss	Unmethylated	Variant lost	NO
		PUB-1JPYH	48	90		Variant lost	CN neutral	Unmethylated	Variant lost	NO
		PUB-OPVC8	79	50	c.540+1G>A p.?	No coverage	Allelic imbalance, CN loss	Unmethylated	WT lost^	YES
WRAP53	4	PUB-EJ4NC	65	68	c.1225C>T p.(Arg409Ter)	Variant lost	Allelic imbalance, CN loss	Unmethylated	Variant lost^	NO
		PUB-WR42I	44	No data	c.682delG p.(Asp228IlefsTer7)	Not performed	Not available	Unmethylated	Variant lost^	NO
		PUB-5JKRY	63	No data	c.910dupT p.(Ser304PhefsTer53)	Not performed	Not available	Unmethylated	Variant lost^	NO
		PUB-FMQOW	60	No data	c.18_19delTC p.(Gln7ThrfsTer27)	Not performed	Not available	Unmethylated	Variant lost^	NO
STARD6	2	PUB-XCS9D	61	36	c.545C>G p.(Ser182Ter)	Heterozygous	CN neutral	Unmethylated	Heterozygous	NO
		PUB-BGL6F	59	85	c.58_59delGA p.(Asp20TyrfsTer8)	WT lost	Homozygous copy loss	Unmethylated	WT lost^	YES
LLGL2	3	PUB-C7OZT	78	40	c.2008delG p.(Ala670LeufsTer54)	Variant lost	CN gain, LoH	Homozygous methylation	Variant lost^ with promoter methylation and amplification of WT allele	YES
		PUB-EJ4NC	65	68	c.2869C>T p.(Arg957Ter)	WT lost	Allelic imbalance	Not performed	WT lost	YES
		PUB-LKOD9	64	62		WT lost	High copy gain, LoH	Not performed	WT lost with amplification of variant allele	YES
CCDC88B	3	PUB-U7P7F	43	31	c.898C>T p.(Gln300Ter)	Heterozygous	CN neutral	PCR failed	Heterozygous	NO
		PUB-WF2F5	63	73	c.3834-1G>C p.?	Heterozygous	Allelic imbalance	PCR failed	Heterozygous	NO
		PUB-J3ODA	66	Low*	c.1028delT p.(Leu343ArgfsTer100)	Heterozygous	CN neutral	PCR failed	Heterozygous	NO
FBLIM1	2	PUB-58A4L	63	73	c.1022_1031delACAGG GCTGG p.(Tyr341CysfsTer40)	Heterozygous	Allelic imbalance, CN gain	PCR failed	Heterozygous	NO
		PUB-IXQKT	70	68	c.1078C>T p.(Arg360Ter)	Heterozygous	CN neutral	PCR failed	Heterozygous	NO
IFIT2	3	PUB-9XXOO	42	81	c.325C>T p.(Arg109Ter)	WT lost	Allelic imbalance, CN loss	No CpG island	WT lost	YES
		PUB-AF099	70	38		Heterozygous	CN gain	No CpG island	Heterozygous^	NO
		PUB-SV5CQ	69	High*		Heterozygous	CN gain	No CpG island	Heterozygous	NO

MIPOL1	3	PUB-SUGLY	73	59	c.182C>G p.(Ser61Ter)	Heterozygous	CN neutral	Heterozygous methylation	Heterozygous^ with promoter methylation of one allele (unknown which allele is methylated)	NO$
		PUB-NFUNH	61	High*	c.1192C>T p.(Arg398Ter)	Heterozygous	CN neutral	Homozygous methylation	Heterozygous with promoter methylation of WT allele	YES
		PUB-HLQJ5	82	35	c.1262+1delG p.?	Heterozygous	Allelic imbalance	Homozygous methylation	Heterozygous with promoter methylation of WT allele	YES
CCDC14	6	PUB-IBX33	58	39	c.139C>T p.(Arg47Ter)	Heterozygous	CN gain	Unmethylated	Heterozygous	NO
		PUB-ROW8I	57	High*	c.1486C>T p.(Gln496Ter)	Heterozygous	CN neutral	Unmethylated	Heterozygous	NO
		PUB-2HQH8	71	High*		Variant lost	Allelic imbalance, CN gain	Not performed	Variant lost	NO
		PUB-HLQJ5	82	35		WT lost	Allelic imbalance, CN gain	Unmethylated	Heterozygous with amplification of variant allele	NO
		PUB-PRTO5	66	59		Heterozygous	CN gain	Unmethylated	Heterozygous	NO
		PUB-DM5J4	49	42		Heterozygous	Allelic imbalance, CN gain	Unmethylated	Heterozygous	NO
TTC24	2	PUB-IFNQ0	65	39	c.1690delA p.(Ser564AlafsTer?)	Heterozygous	CN neutral	Not performed	Heterozygous	NO
		PUB-QY8W8	54	106	c.343C>T p.(Arg115Ter)	Heterozygous	CN neutral	Not performed	Heterozygous	NO
SLC38A8	2	PUB-SWU2E	65	High*	c.697G>T p.(Glu233Ter)	Heterozygous	CN loss	No CpG island	Heterozygous	NO
		PUB-CX65G	66	45		WT lost	LoH	No CpG island	WT lost	YES
CARMIL2	2	PUB-EPBGT	82	27	c.1615_1616delCT p.(Leu539GlyfsTer52)	Variant lost	Allelic imbalance, CN loss	Unmethylated	Variant lost	NO
		PUB-9ZIXX	47	High*	c.4075C>T p.(Arg1359Ter)	Heterozygous	CN neutral	Not performed	Heterozygous	NO
ANKAR	5	PUB-BFSYR	51	96	c.2853_2857delTAAAT p.(Lys952SerfsTer13)	Heterozygous	Allelic imbalance, CN gain	Unmethylated	Heterozygous with amplification of WT allele	NO
		PUB-8U010	74	50	c.3059_3062delAGGA p.(Lys1020ThrfsTer22)	Heterozygous	Allelic imbalance, CN gain	Not performed	Heterozygous with amplification of variant allele	NO
		PUB-5IGWB	49	51		Heterozygous	CN gain	Not performed	Heterozygous	NO
		PUB-88NOV	59	58	c.3019delA p.(Met1007CysfsTer10)	Heterozygous	CN gain	Unmethylated	Heterozygous	NO

		PUB-WANJC	56	48	c.3301-1G>A p.?	Heterozygous	Allelic imbalance, CN gain	Unmethylated	Heterozygous	NO
SCYL3	3	PUB-EVZW5	70	58	c.1474G>A p.?	Heterozygous	CN neutral	Homozygous methylation	Heterozygous with promoter methylation of WT allele	YES
		PUB-9SR2V	55	89	c.1444C>T p.(Arg482Ter)	WT lost	LoH	Not performed	WT lost	YES
		PUB-9ZUZK	68	47		Heterozygous	CN gain	Sanger failed	Heterozygous	NO
MMAA	2	PUB-M4AJ2	65	32	c.439+4_439+7delAGTC p.?	Heterozygous	CN neutral	Not performed	Heterozygous	NO
		PUB-2TCR0	54	61	c.433C>T p.(Arg145Ter)	Heterozygous	CN neutral	Not performed	Heterozygous	NO
ZNF418	2	PUB-ATZ8R	53	76	c.1168C>T p.(Arg390Ter)	Heterozygous	Allelic imbalance, CN gain	Heterozygous methylation	Heterozygous with amplification of WT allele and promoter methylation of one allele (unknown which allele is methylated)	NO$
		PUB-YS9UF	58	High*	c.302_303delAG p.(Gln101ArgfsTer12)	Heterozygous	CN gain	Heterozygous methylation	Heterozygous with promoter methylation of one allele (unknown which allele is methylated)	NO$
USP50	4	PUB-W97N2	45	High*	c.479delG p.(Gly160AspfsTer33)	Heterozygous	CN neutral	No CpG island	Heterozygous	NO
		PUB-QT31P	52	92	c.162C>A p.(Cys54Ter)	Variant lost	Allelic imbalance, CN loss	No CpG island	Variant lost	NO
		PUB-HLQJ5	82	35	c.829delA p.(Arg277GlyfsTer3)	Heterozygous	CN neutral	No CpG island	Heterozygous	NO
		PUB-PRTO5	66	59		Variant lost	Allelic imbalance, CN loss	No CpG island	Variant lost	NO
RAD1	3	PUB-ELA90	47	73	c.168_172delAAAGT p.(Lys57CysfsTer16)	Heterozygous	CN gain	Unmethylated	Variant lost^	NO
		PUB-KTLJF	65	79		Heterozygous	Allelic imbalance, CN gain	Unmethylated	Heterozygous^	NO
		PUB-JTQEO	58	67	c.325C>T p.(Arg109Ter)	Heterozygous	Allelic imbalance, CN gain	Unmethylated	WT lost^	YES
RASSF7	3	PUB-8U010	74	50	c.1030C>T p.(Arg344Ter)	Variant lost	CN neutral	PCR failed	Variant lost	NO

		PUB-YKQRQ	64	49	c.1A>G p.(Met1?)	Variant lost	CN loss, LoH	PCR failed	Variant lost	NO
		PUB-JNKSU	64	91	c.216_220dupCTGCG p.(Gly74AlafsTer13)	Variant lost	Allelic imbalance	PCR failed	Variant lost	NO
LRRC56	1	PUB-K56S6	65	39	c.625-2A>C p.?	Heterozygous	CN neutral	Unmethylated	Heterozygous	NO
HARS2	2	PUB-IBW33	53	68	c.125T>G p.(Ley42Ter)	WT lost	Allelic imbalance, CN loss	Unmethylated	WT lost	YES
		PUB-FXXFL	62	23	c.324T>G p.(Tyr108Ter)	Heterozygous	CN neutral	Heterozygous methylation	Heterozygous with promoter methylation of one allele (unknown which allele is methylated)	NO$
PRKACG	2	PUB-ZPSA2	60	47	c.-5_1dupCCGCCA p.(Phe1_?)	Heterozygous	CN gain	Sanger failed	Heterozygous	NO
		PUB-SKYSL	70	86	c.19A>T p.(Lys7Ter)	Heterozygous	CN gain	Sanger failed	Heterozygous	NO
CDKL3	2	PUB-FWG1Q	41	71	c.924delA p.(Val309SerfsTer4)	Variant lost	Allelic imbalance, CN loss	PCR failed	Variant lost	NO
		PUB-LVJVL	83	57	c.554G>A p.(Trp185Ter)	WT lost	CN loss, LoH	Unmethylated	WT lost	YES
VSIG1	1	PUB-9ZUZK	68	47	c.1211C>G p.(Ser404Ter)	WT lost	CN loss, LoH	No CpG island	WT lost	YES
ZNF616	3	PUB-A6JHW	41	29	c.610C>T p.(Gln204Ter)	Heterozygous	CN neutral	Not performed	Heterozygous	NO
		PUB-JOPWC	74	82	c.13-1G>A p.?	Heterozygous	CN neutral	Not performed	Heterozygous	NO
		PUB-0GFYL	65	66		Heterozygous	CN neutral	Not performed	Heterozygous	NO
ANKRD18A	3	PUB-MWTHR	58	33	c.601A>T p.(Arg201Ter)	Variant lost	Allelic imbalance, CN loss	PCR failed	Variant lost	NO
		PUB-QJ5BB	83	54	c.793C>T p.(Arg265Ter)	Variant lost	Allelic imbalance, CN loss	PCR failed	Variant lost	NO
		PUB-5L8T4	72	42		Variant lost	Allelic imbalance	PCR failed	Variant lost	NO
FAM216A	2	PUB-FWG1Q	41	71	c.436+2C>T p.?	Variant lost	CN loss, LoH	Unmethylated	Variant lost	NO
		PUB-B9PEL	68	64		WT lost	Allelic imbalance	Sanger failed	WT lost	YES
TBXAS1	1	PUB-CX65G	66	45	c.240-1G>T p.?	Heterozygous	Allelic imbalance, CN gain	PCR failed	Heterozygous	NO

CDH23	2	PUB-7IV8M	49	80	c.3006delC p.(Ser1003ProfsTer5)	Heterozygous	Allelic imbalance	Heterozygous methylation	Heterozygous with promoter methylation of one allele (unknown which allele is methylated)	NO[$]
		PUB-COP45	40	65	c.3109A>T p.(Lys1037Ter)	Heterozygous	CN gain	Homozygous methylation	Heterozygous with promoter methylation of WT allele	YES
LTBP1	1	PUB-EOVJJ	65	70	c.864-1G>T p.?	Heterozygous	CN neutral	Homozygous methylation	Heterozygous with promoter methylation of WT allele	YES
ADGRD1	1	PUB-S3571	64	49	c.2T>C p.(Met1?)	Heterozygous	CN gain	No CpG island	Heterozygous	NO
PLEKHA4	2	PUB-I67M5	56	30	c.2306delC p.(Pro769LeufsTer67)	Variant lost	Allelic imbalance, CN gain	No CpG island	Variant lost	NO
		PUB-3K4PE	52	Low*	c.13C>T p.(Arg5Ter)	Heterozygous	CN neutral	No CpG island	Heterozygous	NO
DLGAP5	1	PUB-YU7VB	58	72	c.2112-1G>A p.?	Heterozygous	Allelic imbalance, CN gain	Not performed	Heterozygous	NO

Genes in each group are ordered according to gnomAD rank (see Table 1).
WT: wildtype.
CN: copy number.
[a]ENST00000278616.4:c.2950C>A; ENSP00000278616.4:p.(Gln984Lys).
[b]ENST00000355112.3:c.1515G>A; ENSP00000347232.3:p.(Trp505Ter).
*Quantitative HRD scoring not possible (see Methods); qualitative categorisation performed based on visual inspection of the tumour log_2 CNV profile.
[$]Based on additional bisulphite sequencing data showing presence of gene promoter methylation in normal tissues and/or tumours (see Supplementary Table 4).
[#]Extra HGSOC tumour from known *PALB2* germline LoF variant carrier (not from discovery cohort).
[%]Annotated to Ensembl canonical transcript and protein sequence (see Supplementary Table 5).
^Confirmed by Sanger sequencing.
[@]Assuming somatic stop-gain variant is in WT allele.

Supplementary Table 4

Bisulphite sequencing results for HGSOC tumours with no germline or somatic LoF variants within the corresponding gene, including available ENCODE data from the UCSC Genome Browser[1-3].

KEY

Samples with no somatic or germline LoF variants in that gene (see Supplementary Data)
Genes where bisulphite sequencing failed for all samples
Genes with no evidence of promoter methylation in successfully sequenced samples
Genes with evidence of promoter methylation in one or more sequenced samples

Gene of Interest	ENCODE RRBS Overall*	ENCODE RRBS Fibroblasts and EOC Cell Lines*	ENCODE Methyl 450K Bead Array Overall*	ENCODE Methyl 450K Bead Array Fibroblasts and EOC Cell Lines*	Sample ID	Bisulphite Sequencing Results
ANKAR	No data	No data	Unmethylated	Unmethylated in both	PUB-BFSYR	Unmethylated
					PUB-88NOV	Unmethylated
					PUB-WANJC	Unmethylated
ANKRD18A	Mixed (50-100% methylation in certain cell lines)	0% methylation in both	Partially methylated	Partially methylated in both	PUB-MWTHR	PCR failed
					PUB-QJ5BB	PCR failed
					PUB-5L8T4	PCR failed
ATM	No data	No data	Unmethylated	Unmethylated in both	PUB-WR42I	Unmethylated
					PUB-H03DK	Unmethylated
CARMIL2	Mixed (50-100% methylation in certain cell lines)	0-50% methylation in EOC cells and fibroblasts	Partially methylated	Partially methylated in both	PUB-EPBGT	Unmethylated
CCDC14	0% methylation (all cell lines)	0% methylation in both	Unmethylated	Unmethylated in both	PUB-IBX33	Unmethylated
					PUB-ROW8I	Unmethylated
					PUB-HLQJ5	Unmethylated
					PUB-PRTO5	Unmethylated
					PUB-DM5J4	Unmethylated
CCDC88B	No data	No data	Partially methylated	No data	PUB-U7P7F	PCR failed
					PUB-WF2F5	PCR failed
					PUB-J3ODA	PCR failed
CDH23	Mixed (100% methylation in several cell lines)	0% methylation in both	Partially methylated	Unmethylated in both	PUB-7IV8M	Heterozygous methylation
					PUB-COP45	Homozygous methylation
					PUB-31XA8	Unmethylated
					PUB-EVZW5	Heterozygous methylation
					PUB-L09J6	Unmethylated
					PUB-PRTO5	Homozygous methylation
CDKL3	0% methylation (all cell lines)	0% methylation in both	Unmethylated	Unmethylated in both	PUB-FWG1Q	PCR failed
					PUB-LVJVL	Unmethylated
CPT1B	No data	No data	Unmethylated	Unmethylated in both	PUB-L09J6	Sanger failed
DLGAP5	No data	No data	Unmethylated	Unmethylated in both	PUB-26MQV	PCR failed
FAM216A	0% methylation (all cell lines)	0% methylation in both	Unmethylated	Unmethylated in both	PUB-194H5	Sanger failed
					PUB-FWG1Q	Unmethylated
					PUB-B9PEL	Sanger failed

FANCM	0% methylation (all cell lines with data, CpG island only partially covered)	0% methylation in both	Unmethylated	Unmethylated in both	PUB-WR42I	Unmethylated
					PUB-M8DQW	Unmethylated
					PUB-7HWX3	Unmethylated
					PUB-Q6QAX	Unmethylated
FBLIM1	Mixed (100% methylation in several cell lines)	0% methylation in both	Methylated in several cell lines	Unmethylated in both	PUB-58A4L	PCR failed
					PUB-IXQKT	PCR failed
GPALPP1	0% methylation (all cell lines)	0% methylation in both	Unmethylated	Unmethylated in both	PUB-25VQV	Unmethylated
					PUB-1JPYH	Unmethylated
					PUB-OPVC8	Unmethylated
HARS2	0% methylation (all cell lines)	0% methylation in both	Unmethylated	Unmethylated in both	PUB-IBW33	Unmethylated
					PUB-FXXFL	Heterozygous methylation
					PUB-EVZW5	Heterozygous methylation
					PUB-L09J6	Heterozygous methylation
IMPDH2	No data	No data	Unmethylated	Unmethylated in both	PUB-WF2F5	Unmethylated
LLGL2	0% methylation (all cell lines except one)	0% methylation in both	Unmethylated	Unmethylated in both	PUB-C7OZT	Homozygous methylation
					PUB-L09J6	Heterozygous methylation
					PUB-FXXFL	Unmethylated
					PUB-LVJVL	Homozygous methylation
LOXL2	Mixed (50-100% methylation in certain cell lines)	0-25% methylation in EOC cells; 0% methylation in fibroblasts	Partially methylated (certain cell lines)	Unmethylated in both	PUB-LYFY5	Unmethylated
					PUB-PWBKB	Unmethylated
LRRC56	Mixed (0-50% methylation in certain cell lines with only three fully methylation)	0-50% methylation in EOC cells; 0-25% methylation in fibroblasts	Partially methylated	Partially-to-fully methylated in EOC; partially methylated in fibroblasts	PUB-K56S6	Unmethylated
LTBP1	0% methylation (all cell lines except one)	0% methylation in both	Unmethylated	Unmethylated in both	PUB-Z7JTY	Homozygous methylation
					PUB-EOVJJ	Homozygous methylation
					PUB-EVZW5	Heterozygous methylation
MAP6D1	Mixed (0-50% methylation in certain cell lines with only five fully methylation)	50% methylation in EOC cells at 3` end; 0% methylation in fibroblasts	Partially methylated (certain cell lines)	Partially methylated in EOC cells; unmethylated in fibroblasts	PUB-R0JXJ	Heterozygous methylation
					PUB-HLQJ5	Homozygous methylation
					PUB-PRTO5	Heterozygous methylation
					PUB-L09J6	Heterozygous methylation

MIPOL1	Mixed (0-50% methylation in certain cell lines with only three fully methylation)	0% methylation in both	Partially methylated (certain cell lines)	Unmethylated in both	PUB-SUGLY	Heterozygous methylation
					PUB-NFUNH	Homozygous methylation
					PUB-HLQJ5	Homozygous methylation
					PUB-L09J6	Unmethylated
					PUB-PRTO5	Heterozygous methylation
					PUB-EVZW5	Unmethylated
					PUB-EJ4NC	Heterozygous methylation
					PUB-OPVC8	Unmethylated
MRE11A	No data	No data	Unmethylated	Unmethylated in both	PUB-J9S7G	Unmethylated
					PUB-EVZW5	Unmethylated
					PUB-ROW8I	Unmethylated
RAD1	0% methylation (all cell lines)	0% methylation in both	Unmethylated	Unmethylated in both	PUB-ELA90	Unmethylated
					PUB-KTLJF	Unmethylated
					PUB-JTQEO	Unmethylated
RASSF7	0% methylation (all cell lines)	0% methylation in both	Unmethylated	Unmethylated in both	PUB-8U010	PCR failed
					PUB-YKQRQ	PCR failed
					PUB-JNKSU	PCR failed
RPA3	0% methylation (all cell lines)	0% methylation in both	Unmethylated	Unmethylated in both	PUB-MWTHR	PCR failed
					PUB-UGMNM	PCR failed
SCYL3	No data	No data	Unmethylated	Unmethylated in both	PUB-EVZW5	Homozygous methylation
					PUB-9ZUZK	Sanger failed
					PUB-1JPYH	Homozygous methylation
SLC12A4	Mixed (0-100% methylation in certain cell lines)	0-25% methylation in EOC cells; 0% methylation in fibroblasts	Partially methylated (certain cell lines)	Partially-to-fully methylated in EOC cells and fibroblasts	PUB-KH12P	PCR failed
					PUB-SWU2E	PCR failed
					PUB-YS9UF	PCR failed
STARD6	0% methylation (all cell lines except one)	0% methylation in both	Partially methylated (few cell lines)	No data	PUB-XCS9D	Unmethylated
					PUB-BGL6F	Unmethylated
TBXAS1	0% methylation (all cell lines except one)	0% methylation in both	Unmethylated	Unmethylated in both	PUB-LCVA9	PCR failed
					PUB-CX65G	PCR failed
WRAP53	0% methylation (all cell lines)	0% methylation in both	Unmethylated	Unmethylated in both	PUB-EJ4NC	Unmethylated
ZBTB45	0% methylation (all cell lines)	0% methylation in both	Unmethylated	Unmethylated in both	PUB-7V374	Unmethylated
					PUB-BT3YN	Unmethylated
ZCCHC4	No data (3` end in gene coding region 0% methylation)	No data (3` end in gene coding region 0% methylation)	Unmethylated	Unmethylated in both	PUB-XCS9D	Sanger failed
					PUB-TENNY	Sanger failed
					PUB-FWG1Q	Sanger failed
					PUB-7GYYG	Sanger failed
					PUB-ZRDAA	Sanger failed
					PUB-EZ87I	Sanger failed

ZNF418	Mixed (50-100% methylation in certain cell lines)	0-25% methylation in EOC cells and fibroblasts	Methylated/partially methylated in several cell lines	Partially methylated in EOC cells; mostly unmethylated in fibroblasts	PUB-ATZ8R	Heterozygous methylation
					PUB-YS9UF	Heterozygous methylation
					PUB-EJ4NC	Heterozygous methylation

ENCODE data sourced from the UCSC Genome Browser[1-3].
RRBS: reduced representation bisulphite sequencing.
EOC: epithelial ovarian carcinoma.

Supplementary Table 5

List of Ensembl canonical gene, transcript and protein sequences used for variant annotation.

Gene Symbol	Gene ID	Transcript ID	Protein ID
ADGRD1	ENSG00000111452	ENST00000261654	ENSP00000261654
ANKAR	ENSG00000151687	ENST00000520309	ENSP00000427882
ANKRD18A	ENSG00000180071	ENST00000399703	ENSP00000382610
ATM	ENSG00000149311	ENST00000278616	ENSP00000278616
BLM	ENSG00000197299	ENST00000355112	ENSP00000347232
CARMIL2	ENSG00000159753	ENST00000334583	ENSP00000334958
CCDC14	ENSG00000175455	ENST00000433542	ENSP00000395706
CCDC88B	ENSG00000168071	ENST00000356786	ENSP00000349238
CDH23	ENSG00000107736	ENST00000398788	ENSP00000381768
CDKL3	ENSG00000006837	ENST00000265334	ENSP00000265334
CPT1B	ENSG00000205560	ENST00000360719	ENSP00000353945
DLGAP5	ENSG00000126787	ENST00000247191	ENSP00000247191
ERCC3	ENSG00000163161	ENST00000285398	ENSP00000285398
FAM216A	ENSG00000204856	ENST00000377673	ENSP00000366901
FANCM	ENSG00000187790	ENST00000267430	ENSP00000267430
FBLIM1	ENSG00000162458	ENST00000441801	ENSP00000416387
GPALPP1	ENSG00000133114	ENST00000361121	ENSP00000355211
HARS2	ENSG00000112855	ENST00000230771	ENSP00000230771
IFIT2	ENSG00000119922	ENST00000371826	ENSP00000360891
IMPDH2	ENSG00000178035	ENST00000326739	ENSP00000321584
LLGL2	ENSG00000073350	ENST00000392550	ENSP00000376333
LOXL2	ENSG00000134013	ENST00000389131	ENSP00000373783
LRRC56	ENSG00000161328	ENST00000270115	ENSP00000270115
LTBP1	ENSG00000049323	ENST00000404816	ENSP00000386043
MAP6D1	ENSG00000180834	ENST00000318631	ENSP00000314560
MIPOL1	ENSG00000151338	ENST00000327441	ENSP00000333539
MMAA	ENSG00000151611	ENST00000281317	ENSP00000281317
MRE11A	ENSG00000020922	ENST00000323929	ENSP00000325863
PALB2	ENSG00000083093	ENST00000261584	ENSP00000261584
PLEKHA4	ENSG00000105559	ENST00000263265	ENSP00000263265
PRKACG	ENSG00000165059	ENST00000377276	ENSP00000366488
RAD1	ENSG00000113456	ENST00000382038	ENSP00000371469
RASSF7	ENSG00000099849	ENST00000397583	ENSP00000380713
RPA3	ENSG00000106399	ENST00000223129	ENSP00000223129
SCYL3	ENSG00000000457	ENST00000367772	ENSP00000356746
SLC12A4	ENSG00000124067	ENST00000422611	ENSP00000395983
SLC38A8	ENSG00000166558	ENST00000299709	ENSP00000299709
SORD	ENSG00000140263	ENST00000267814	ENSP00000267814
SSX3	ENSG00000165584	ENST00000298396	ENSP00000298396
STARD6	ENSG00000174448	ENST00000581310	ENSP00000462349
TBXAS1	ENSG00000059377	ENST00000416849	ENSP00000389414
TTC24	ENSG00000187862	ENST00000368236	ENSP00000357219
USP50	ENSG00000170236	ENST00000532404	ENSP00000434676
VSIG1	ENSG00000101842	ENST00000415430	ENSP00000402219
WRAP53	ENSG00000141499	ENST00000316024	ENSP00000324203
ZBTB45	ENSG00000119574	ENST00000594051	ENSP00000469089
ZCCHC4	ENSG00000168228	ENST00000302874	ENSP00000303468
ZNF418	ENSG00000196724	ENST00000396147	ENSP00000379451
ZNF616	ENSG00000204611	ENST00000600228	ENSP00000471000

Supplementary Figures

Supplementary Figure 1

Flowcharts showing filtering performed on the (a) WES and (b) WGS tumour sequencing data to generate somatic variants for downstream analysis (see Methods and Supplementary Methods). Differences for the *PALB2* sample (PUB-XXXXX) without accompanying germline data are noted where relevant. Created using Microsoft PowerPoint.

(a)

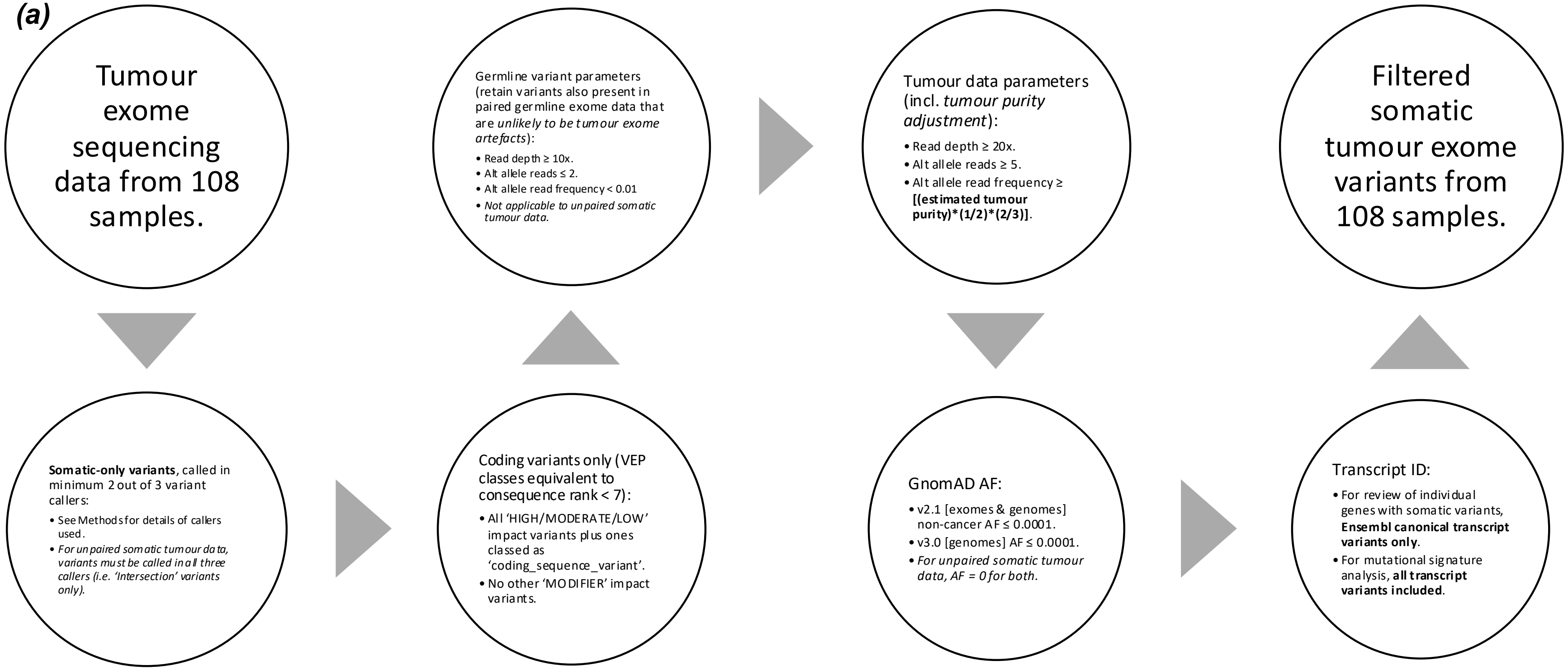

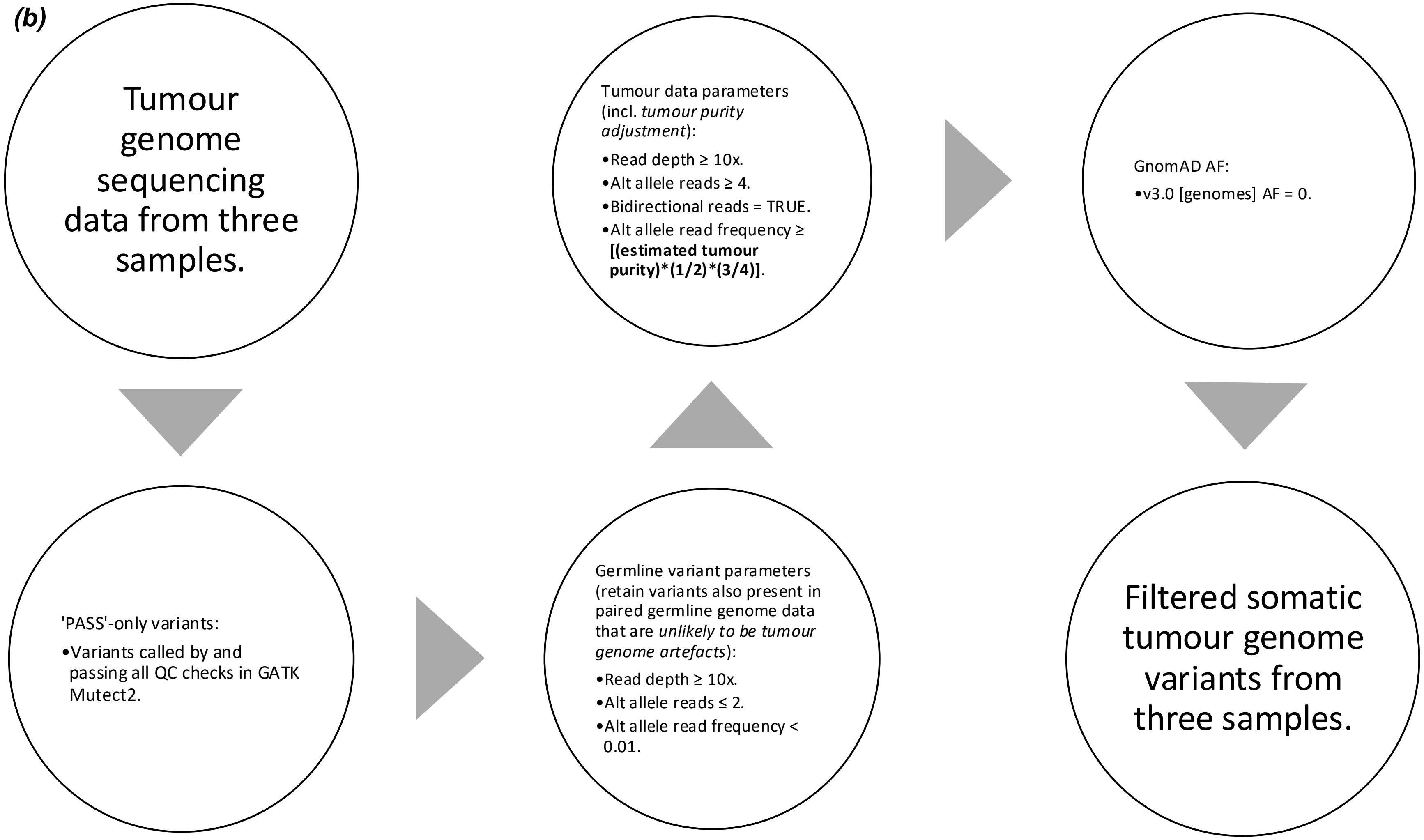
(b)
Tumour genome sequencing data from three samples.
'PASS'-only variants:
•Variants called by and passing all QC checks in GATK Mutect2.
Germline variant parameters (retain variants also present in paired germline genome data that are *unlikely to be tumour genome artefacts*):
•Read depth ≥ 10x.
•Alt allele reads ≤ 2.
•Alt allele read frequency < 0.01.
Tumour data parameters (incl. *tumour purity adjustment*):
•Read depth ≥ 10x.
•Alt allele reads ≥ 4.
•Bidirectional reads = TRUE.
•Alt allele read frequency ≥ **[(estimated tumour purity)*(1/2)*(3/4)]**.
GnomAD AF:
•v3.0 [genomes] AF = 0.
Filtered somatic tumour genome variants from three samples.

Supplementary Figure 2

Bisulphite sequencing chromatograms (forward direction) of *LLGL2* promoter CpG island using tumour DNA from individual with germline *LLGL2* LoF variant and intact WT allele in the tumour (see Table 2 and Supplementary Table 3), alongside female and CpG-methylated reference control DNA. Visualised using Geneious 8.1.9[4].

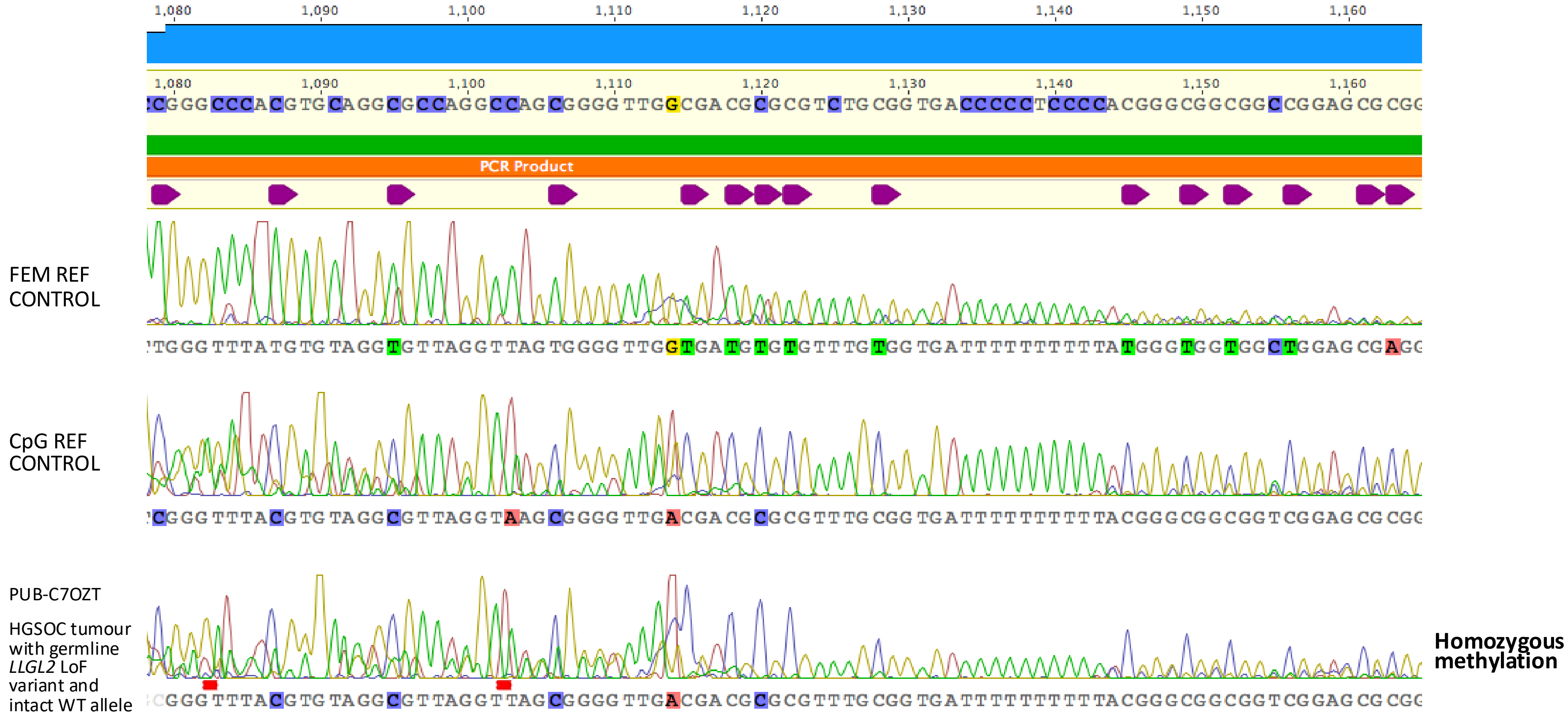

Supplementary Figure 3

HGSOC tumour IHC and staining pattern using a primary monoclonal antibody at 1:200 dilution to LLGL2 protein (see Methods), on (a) three sections from tumours harbouring at least one intact *LLGL2* WT allele, and (b) three sections from tumours with biallelic *LLGL2* inactivation (see Table 2). Visualised using OlyVIA 2.9[5].

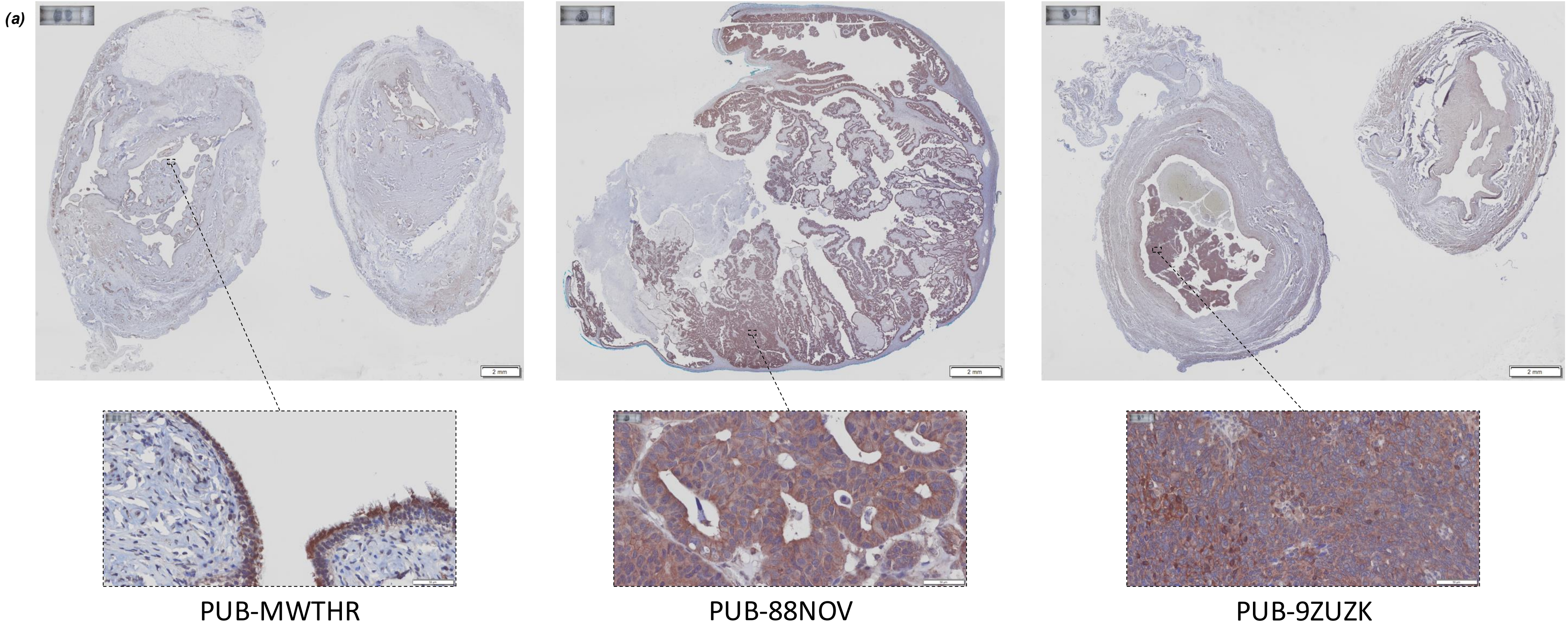

Diffuse, strongly positive, cytoplasmic.

(b)

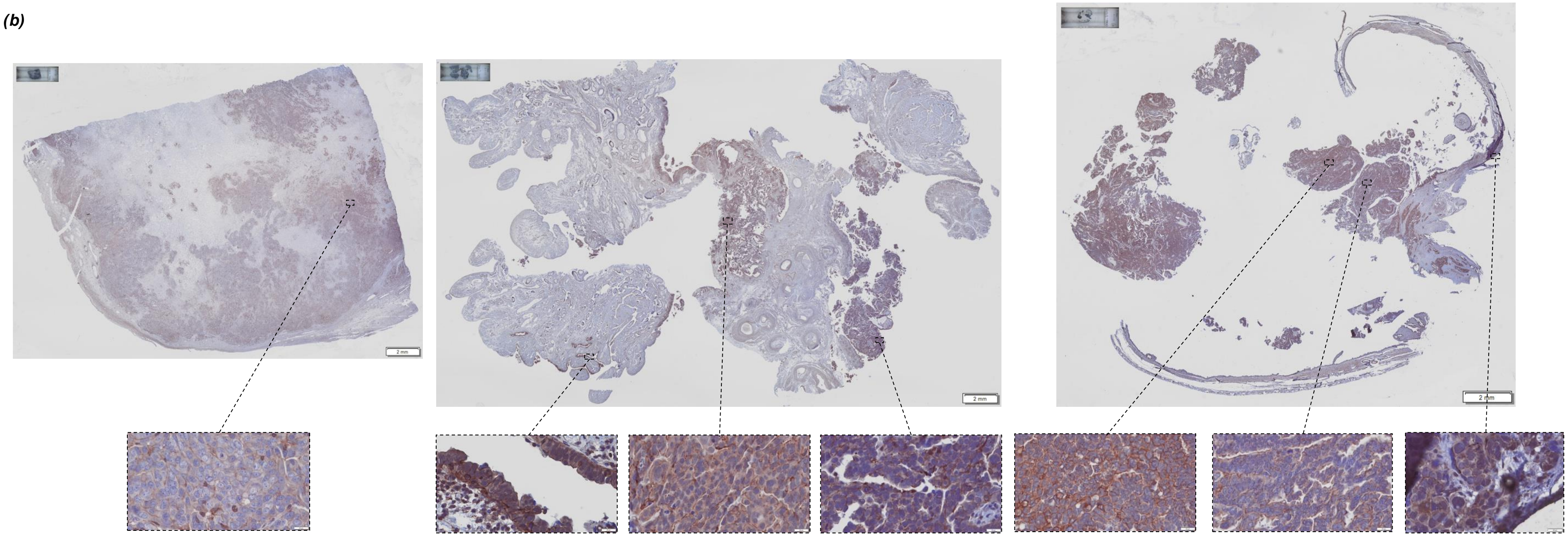

PUB-EJ4NC

Diffuse, very weakly positive, peri-membranous.

PUB-C70ZT

PUB-LKOD9

Patchy/variable, weakly positive, peri-membranous.

Supplementary Figure 4
SIGNAL fitted mutational signatures (using the ovary-specific set and SIGNAL FitMS algorithm[6]) for WGS somatic HGSOC tumour variants from *LLGL2*-inactivated tumours, with number of filtered somatic variants used for fitting and calculated HRD scores (see Methods). Signatures that did not pass the sparsity threshold and were not called are shaded. Crosses (‘X’) represent median contribution estimates; box plots provide the contribution estimate distribution for each signature (boxes and error bars denote mean +/- interquartile ranges and 95% confidence intervals, respectively); dashed lines represent (dynamic) sparsity filter thresholds. Created using SIGNAL Analyse 2 platform[6].

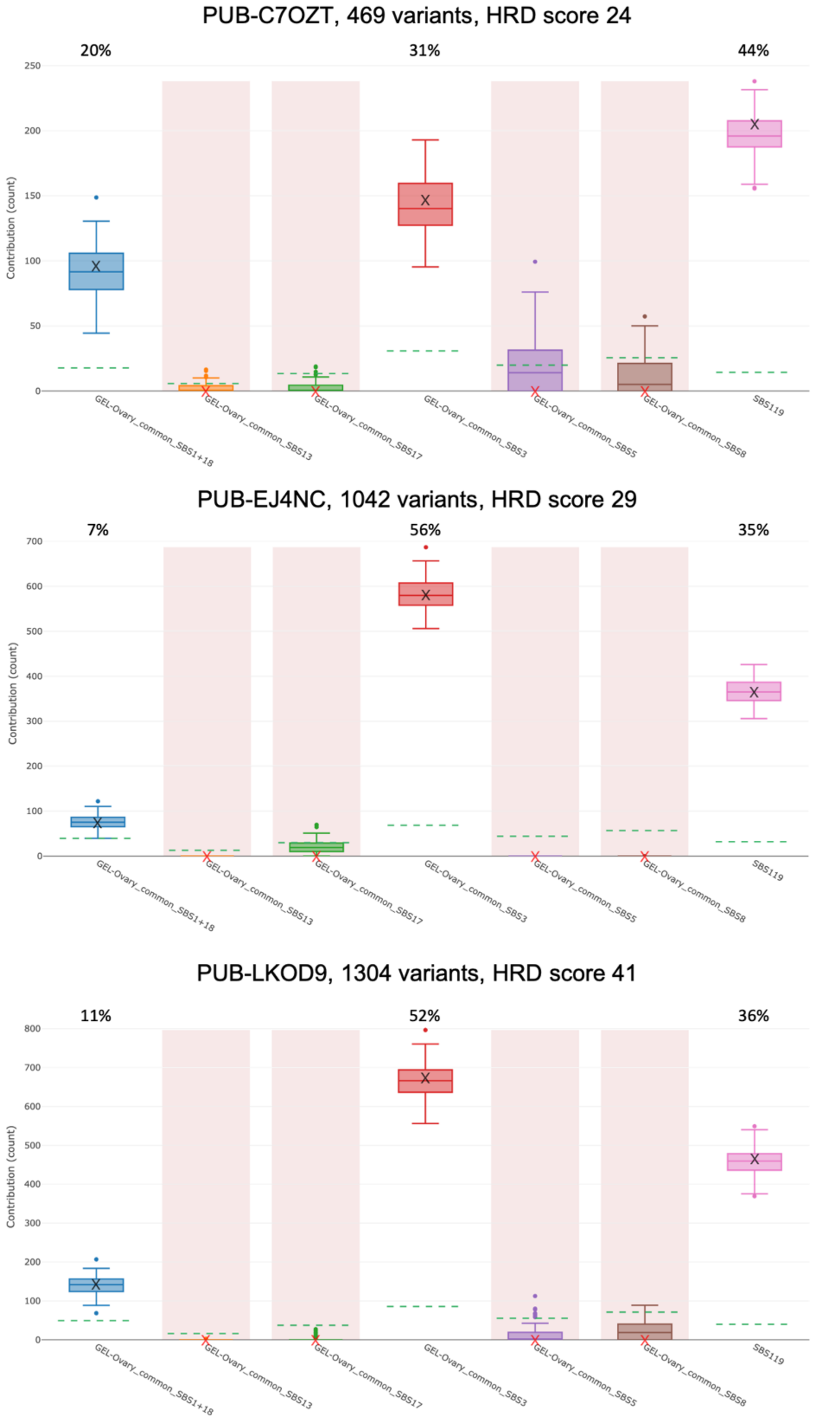

References

1. Rosenbloom, K. R. *et al.* ENCODE whole-genome data in the UCSC Genome Browser. *Nucleic Acids Res,* **38**, D620-5 (2010).

2. Rosenbloom, K. R. *et al.* ENCODE data in the UCSC Genome Browser: year 5 update. *Nucleic Acids Res,* **41**, D56-63 (2013).

3. The ENCODE Project Consortium. An integrated encyclopedia of DNA elements in the human genome. *Nature,* **489**, 57-74 (2012).

4. Geneious v8.1.9. https://www.geneious.com (Biomatters, Auckland, New Zealand, 2016).

5. OlyVIA v2.9. https://www.olympus-lifescience.com/en/downloads/detail-iframe/?0[downloads][id]=847249644 (Evident Corporation, Tokyo, Japan, 2023).

6. Degasperi, A. *et al.* Substitution mutational signatures in whole-genome-sequenced cancers in the UK population. *Science,* **376**, 368 (2022).
